# Engineering of bioactive metal sulfide nanomaterials for cancer therapy

**DOI:** 10.1186/s12951-021-00839-y

**Published:** 2021-03-31

**Authors:** Weidong Fei, Meng Zhang, Xiaoyu Fan, Yiqing Ye, Mengdan Zhao, Caihong Zheng, Yangyang Li, Xiaoling Zheng

**Affiliations:** 1 Department of Pharmacy, Women’s Hospital, Zhejiang University School of Medicine, Hangzhou, 310006 China; 2 School of Pharmacy, Faculty of Medicine and Health, The University of Sydney, Sydney, 2006 Australia; 3 Key Laboratory of Women′s Reproductive Health Research of Zhejiang Province, Women’s Hospital, Zhejiang University School of Medicine, Hangzhou, 310006 China

**Keywords:** Metal sulfide, Nanomaterials, Fabrication, Cancer, Therapy

## Abstract

Metal sulfide nanomaterials (MeSNs) are a novel class of metal-containing nanomaterials composed of metal ions and sulfur compounds. During the past decade, scientists found that the MeSNs engineered by specific approaches not only had high biocompatibility but also exhibited unique physicochemical properties for cancer therapy, such as Fenton catalysis, light conversion, radiation enhancement, and immune activation. To clarify the development and promote the clinical transformation of MeSNs, the first section of this paper describes the appropriate fabrication approaches of MeSNs for medical science and analyzes the features and limitations of each approach. Secondly, we sort out the mechanisms of functional MeSNs in cancer therapy, including drug delivery, phototherapy, radiotherapy, chemodynamic therapy, gas therapy, and immunotherapy. It is worth noting that the intact MeSNs and the degradation products of MeSNs can exert different types of anti-tumor activities. Thus, MeSNs usually exhibit synergistic antitumor properties. Finally, future expectations and challenges of MeSNs in the research of translational medicine are spotlighted.

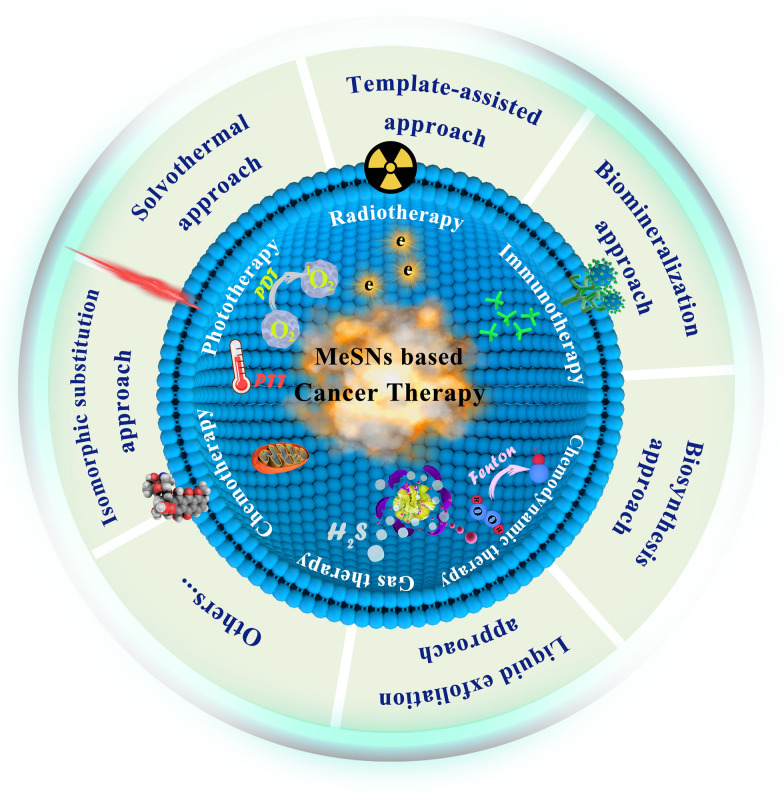

## Introduction

Nowadays, cancers turn out to be the second leading cause of death (after cardiovascular diseases) around the world [1]. Various clinical therapeutic strategies, such as surgery, immunotherapy, chemotherapy, and radiation therapy, have been applied either individually or in combination to treat different types of cancers. Particularly, chemotherapy being a non-invasive approach has attracted great attention due to its advantages such as short recovery time, easy targeting of cancer cells, and high compliance. Metal-containing compounds have been demonstrated as an effective treatment in cancer patients. For instance, lots of platinum (Pt)-containing anti-cancer drugs such as cisplatin, lobaplatin, carboplatin, and oxaliplatin have been applied to the clinic to treat approximately 50–70% of cancers [2]. Gallium is the second metal element used to treat tumors after Pt [3]. Since Hart et al. discovered the anti-tumor activity of gallium nitrate in 1971 [4], it has been continuously studied and applied to the treatment of non-small cell lung cancer, prostate cancer, and breast cancer [5–7]. Metal-containing compounds play important roles in cancer therapy, but the short circulation time, low target selectivity, and systematic toxicity caused by single metal ions have resulted in a wide range of adverse reactions, including hepatotoxicity, gastrointestinal reactions, neurotoxicity, bone marrow suppression, nephrotoxicity, ototoxicity, severe nausea/vomiting and hair loss [8].

**Fig. 1:**
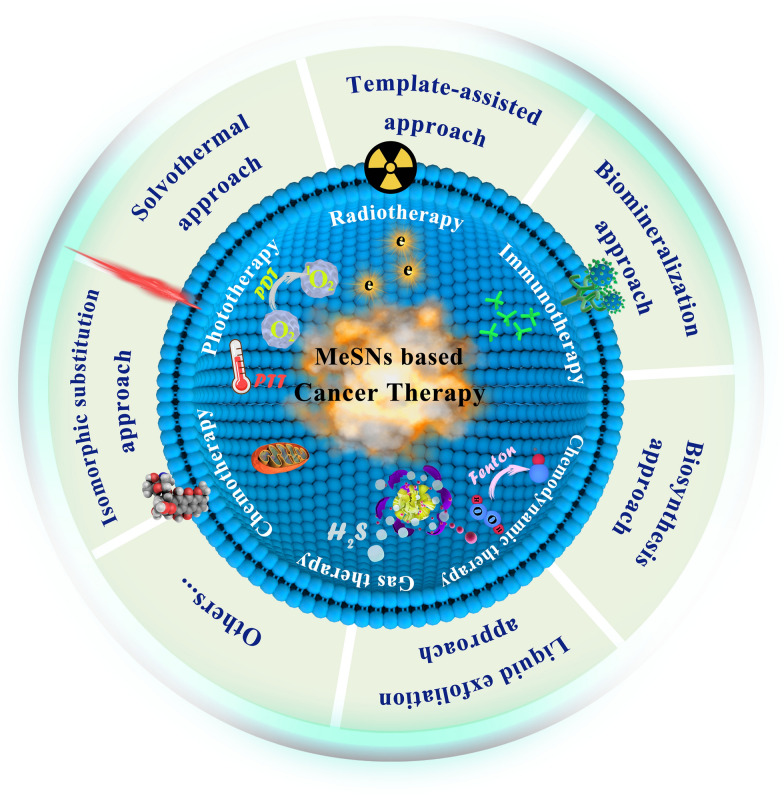
Schematic illustration showing the preparation approaches and antitumor mechanisms of MeSNs

Regarding the above defects of metal compounds, metal-containing bioactive nanomaterials (including metal-organic framework, metal sulfide, metal carbide, metal oxide, etc.) have attracted considerable attention in cancer therapy [9–11]. Specifically, metal sulfide nanomaterials (MeSNs) prepared by specific fabrication approaches (such as solvothermal approach, template-assisted approach, biomineralization approach, isomorphic substitution approach, liquid exfoliation approach, and biosynthesis approach) exhibit special physical and chemical properties, such as Fenton catalysis, light conversion, radiation enhancement and immune activation [12–16]. These are excellent features in the field of tumor treatment [17]. For instance, copper sulfide (CuS) nanoparticles (NPs) have a broad absorption in the near-infrared region (NIR). The aqueous dispersion of the nanoparticles exhibits excellent photothermal conversion efficiency under the laser irradiation at a wavelength of 808 nm. Therefore, it can be used as a photothermal agent against tumors [15, 18, 19]. Some types of MeSNs, such as manganese sulfide (MnS) NPs, iron sulfide (FeS) NPs, and zinc sulfide (ZnS) NPs, can be used as gas therapeutic agents because they can dissociate hydrogen sulfide (H_2_S) gas in the acidic environment of tumors [14, 20, 21]. Although the application of MeSNs as theranostic nanoplatforms has been an area of research for a decade, only a few comprehensive reviews spotlight the recent progress as well as contemporary challenges. To clarify the developing direction and focus of MeSNs in the field of medical science, it is urgent to outline the latest advances of MeSNs in cancer therapy (Fig. 1).

## Engineering of metal sulfide nanomaterials

Various approaches can be used to synthesize MeSNs, such as gas vulcanization approach, wet chemical approach, electrochemical deposition, mechanochemical approach, and pyrolytic approach, etc. [22–26]. However, MeSNs engineered by some types of approaches cannot meet the standards of biomedical applications. For example, the materials prepared by electrochemical deposition and gas vulcanization approach cannot meet the requirement of nanometer-scale and are unstable in aqueous systems. Generally, electrochemical deposition is widely used in energy storage and conversion systems by synthesizing layered and reticulated metal sulfides [25, 27]. While the gas vulcanization approach is famous for its ability to remove heavy metals from waste water or metal scraps [26, 28]. The MeSNs designed for biomedical applications must meet the following three criteria to be highly biocompatible: (i) low toxicity: for example, tellurium is categorized as a nonessential and toxic element, thus tellurium sulfide nanomaterials are not suitable for biomedical applications [29]. In comparison, MeSNs without direct toxicity to normal tissues are more suitable for therapeutic uses [30, 31]; (ii) good dispersibility and high physiological stability: MeSNs upon entering the living body contact the cells and tissues directly; thus, being dispersible, water-soluble, and relatively stable in biosystems are the fundamental properties of MeSNs to elicit anti-tumor effects [22, 32]; (iii) suitable particle size: the altered anatomy of the tumor vessels only allows nanoparticles with a certain size to be extravasated from the circulation into the tumor tissues, where they increase the retention effect due to poor lymphatic drainage as well as the enhanced vascular permeability. Thus, a larger amount of the theranostic agents are delivered to tumors compared to that of the normal tissues [33, 34]. Additionally, renal excretion increases when the diameters of the NPs decrease to ultrasmall range, which further minimize the toxicity of the nanoformulations [35]. Therefore, MeSNs with suitable diameters have optimized biodistribution and better theranostic outcomes. Generally, choosing a suitable preparation approach is vital to obtain bioactive MeSNs for biological applications.

The most convenient approach to prepare MeSNs for medical applications is coprecipitation at room temperature. After mixing sodium sulfide and metal salts in the solvent, metal ions act as central atoms to continuously combine with sulfur ligands and eventually form insoluble precipitates [36]. However, the reaction rate of the coprecipitation approach is slow with low yield, and the morphology and size of the crystalline are poorly controlled. Furthermore, the prepared nanomaterials are more likely to sediment due to their hydrophobic surfaces [37, 38]. These problematic characteristics extremely limit the application of these products in the medical field, which boost the discovery of novel fabrication techniques to develop MeSNs with high biocompatibility and production efficiency. After extensive reviews of various preparation approaches, it was found that MeSNs with relatively high biocompatibility can be produced by the solvothermal approach, biomineralization approach, isomorphic substitution approach, liquid exfoliation approach, template-assisted approach, and biosynthesis approach (Table 1). The hydrophilicity and biocompatibility of MeSNs can be improved by adding hydrophilic organic compounds such as polyvinylpyrrolidone (PVP), chitosan, and protein during the fabrication process [13, 19, 39] or modifying polyethylene glycol (PEG), lipid, or other biocompatible molecules on the outer surface of MeSNs [33, 40, 41]. This section aims to analyze the properties and limitations of different fabrication techniques to guide researchers in choosing a suitable approach.

**Table 1:** Classification of appropriate synthesis approaches of MeSNs for the medical science

	MeSNs	Metal source	Sulfur source	Temperature	Template	Particle size	Ref.
Hydrothermal approach	CuS NPs	CuCl_2_·2H_2_O	Na_2_S	90 °C	NA	Around 10 nm	[44]
	MoS_2_ nanoflakes	Na_2_MoO_4_·2H_2_O	Thiourea	200 °C	NA	200 to 350 nm	[46]
	Ni_9_S_8_ NPs	Ni(NO_3_)_2_·6H_2_O	1-Dodecanethiol	200 °C	NA	About 150 nm	[45]
	Ag_2_S NPs	AgNO_3_	Na_2_S·9H_2_O	50 °C	NA	About 15 nm	[47]
	MoS_2_ nanosheets	(NH_4_)_2_MoO_4_	Thiourea	200 °C	NA	About 150 nm	[142]
Non-aqueous solvothermal approach	CuS nanocrystals	Copper acetylacetonate	Sulfur powder	70 °C	NA	About 8 nm	[52]
	NiS NPs	Nickel acetate tetrahydrate	Thioacetamide	150 °C	NA	247 nm	[48]
	Bi_2_S_3_ nanorods	Bismuth neodecanoate	Thioacetamide	150 °C	NA	100 nm in length and 15 nm in diameter	[49]
	Bi_2_S_3_ nanorods	Bismuth neodecanoate	Thioacetamide	150 °C	NA	About13 nm in diameter and 40 nm in length	[50]
	Bi_2_S_3_ nanorods	Bismuth neodecanoate	Thioacetamide	150 ℃	NA	About 15 nm in diameter and 60 nm in length	[51]
	Bi_2_S_3_ nanorods	Bismuth neodecanoate	Thioacetamide	150 °C	NA	10 nm in diameter and 50 nm in length	[53]
	CuS-ZnS nanocrystals	Cu(acac)_2_/Zn(acac)_2_	1- dodecanethiol	200 °C	NA	About16 nm	[143]
	RuS_1.7_ ND	RuCl_3_ nanodots	Diethyl dithiocarbamate	–	NA	About 70 nm	[98]
Template-assisted approach	CuS NPs	[Cu(NH_3_)_4_]^2+^ solution	Na_2_S·9H_2_O	50–90 °C	Chitosan	5.6 nm	[19]
	CuS NPs	CuCl_2_·2H_2_O	Na_2_S	90 °C	Biopolymer melanin	About 21 nm	[13]
	CuS NPs	CuCl_2_	Na_2_S	75 °C	PVP-K30	200 nm	[39]
	CuS NPs	CuCl_2_	Na_2_S	75 °C	Cetyltrimethylammonium chloride	10 nm	[12]
	Bi_2_S_3_ NPs	Bi(NO_3_)_3_·5H_2_O	Na_2_S·9H_2_O	180 °C	PVP	Around 130 nm in length and 60 nm in width	[89]
	Fe_1 − x_S NPs	Fe(NH_4_)_2_·(SO_4_)_2_	Thioacetamide	180 °C	PVP	20–30 nm	[140]
	ZnS NPs	Zinc acetate dihydrate	Thiourea	125 °C	Silica nanofibres	About 110 nm	[21]
Biomineralization approach	Biological sulfur sources						
	Bi_2_S_3_ NPs	Bi(NO_3_)_3_	BSA	25 °C	BSA	107.6 ± 6.81 nm	[57]
	Bi_2_S_3_ NPs	Bi(NO_3_)_3_	BSA	25 °C	BSA	78.9 nm	[56]
	Bi_2_S_3_ NPs	Bi(NO_3_)_3_	BSA	25 °C	BSA	10 ± 3 nm	[15]
	Bi_2_S_3_ NPs	Bi(NO_3_)_3_	BSA	25 °C	BSA	6.1 ± 0.9 nm	[31]
	Double sulfur sources						
	FeS@BSA NPs	FeCl_2_	Na_2_S	4 °C	BSA	About 50 nm	[14]
	MnS NPs	Mn(NO_3_)_2_	Na_2_S	25 °C	BSA	About 150 nm	[64]
	Mn-CuS NDs	CuCl_2_ and MnCl_2_	Na_2_S·9H_2_O	90 °C	BSA	4.95 nm	[144]
	Co_9_S_8_ NDs	CoCl_2_ or CoSO_4_	Na_2_S	37 °C	BSA	About 14.5 nm	[67]
	ZnS NPs	ZnCl_2_	Thioacetamide	25 °C	BSA	15.9 ± 2.1 nm	[145]
	Ag_2_S nanorods	AgNO_3_	Thioacetamide	25 °C	BSA	65 nm	[65]
	Ag_2_S NPs	AgNO_3_	Thioacetamide	25 °C	BSA	6.98 nm	[66]
Isomorphic substitution approach	Substitute the metal ions						
	CuS nanodots	CuCl_2_	Na_2_S	90 °C	Layered double hydroxide	10 nm CuS nanodots in 120 nm layered double hydroxide	[30]
	Bi_2_S_3_ nanorods	Bi(NO_3_)_3_·5H_2_O	Thiourea and ZnS microspheres	130 °C	ZnS composite microspheres	300–500 nm, 230 nm	[18, 69]
	Substitute the anions						
	Hollow CuS NPs	CuCl_2_·2H_2_O	(NH_4_)_2_ S	RT	CuO NPs	184.2 ± 4.8 nm	[85]
	Hollow CuS NPs	CuCl_2_	Na_2_S	60 °C	CuO NPs	100 nm	[70]
Liquid exfoliation approach	SnS nanosheets	Bulk SnS	Bulk SnS	Ultrasound heat	NA	Less than 50 nm	[72]
	TaS_2_ nanosheets	Raw TaS_2_ materials	Raw TaS_2_ materials	Ultrasound heat	NA	About 110 nm	[40]
	WS_2_ quantum dots	Commercial WS_2_ bulk	Bulk WS_2_	90 °C	NA	20–100 nm	[33, 41]
Wetchemical approach	Fe-doped CaS NPs	FeCl_2_·2H_2_O and CaCl_2_	Na_2_S·9H_2_O	RT	NA	About 47.5 nm	[36]
	ReS_2_ NPs	NaReO_4_	Na_2_S_2_O_3_·5H_2_O	RT	NA	3 ± 0.21 nm	[22]
	Gold-gold sulfide nanoshells	HAuCl_4_	Na_2_S	RT	NA	About 5.4 nm	[55]
Biosynthesis	CdS NPs	Cadmium nitrate	Na_2_S	30 °C	NA	About 15 nm	[81]
	FeS NPs	FeOOH NSs	Endogenous H_2_S	37 °C	NA	About 5 nm	[16]
Mechanochemical approach	ZnS Nanocrystals	Zinc acetate	Na_2_S	Milling heat	NA	614–987 nm	[83]
Pyrolytic approach	ZnS NPs	[Zn(SCN)_2_(2-benzoylpyridine)_2_]	[Zn(SCN)_2_(2-benzoylpyridine)_2_]	620 °C	NA	80–120 nm	[23]

## Solvothermal approach

The solvothermal approach is the most common approach to prepare MeSNs. The fundamental principle is to dissolve metal salts (including inorganic metal salts and organic metal salts) and sulfides into the solvent. After that, the seed will be formed as a coordination complex by combining metal central atoms and sulfide ligands, which will then grow to yield the MeSNs under a hydrothermal environment [42, 43]. This process can be accelerated by raising the temperature and adding organic templates. The solvothermal approach, including the hydrothermal approach and the non-aqueous solvothermal approach, is widely used in the synthesis of MeSNs due to its simplicity, low cost, and short preparation time (Table 1).

### Hydrothermal approach

**Fig. 2:**
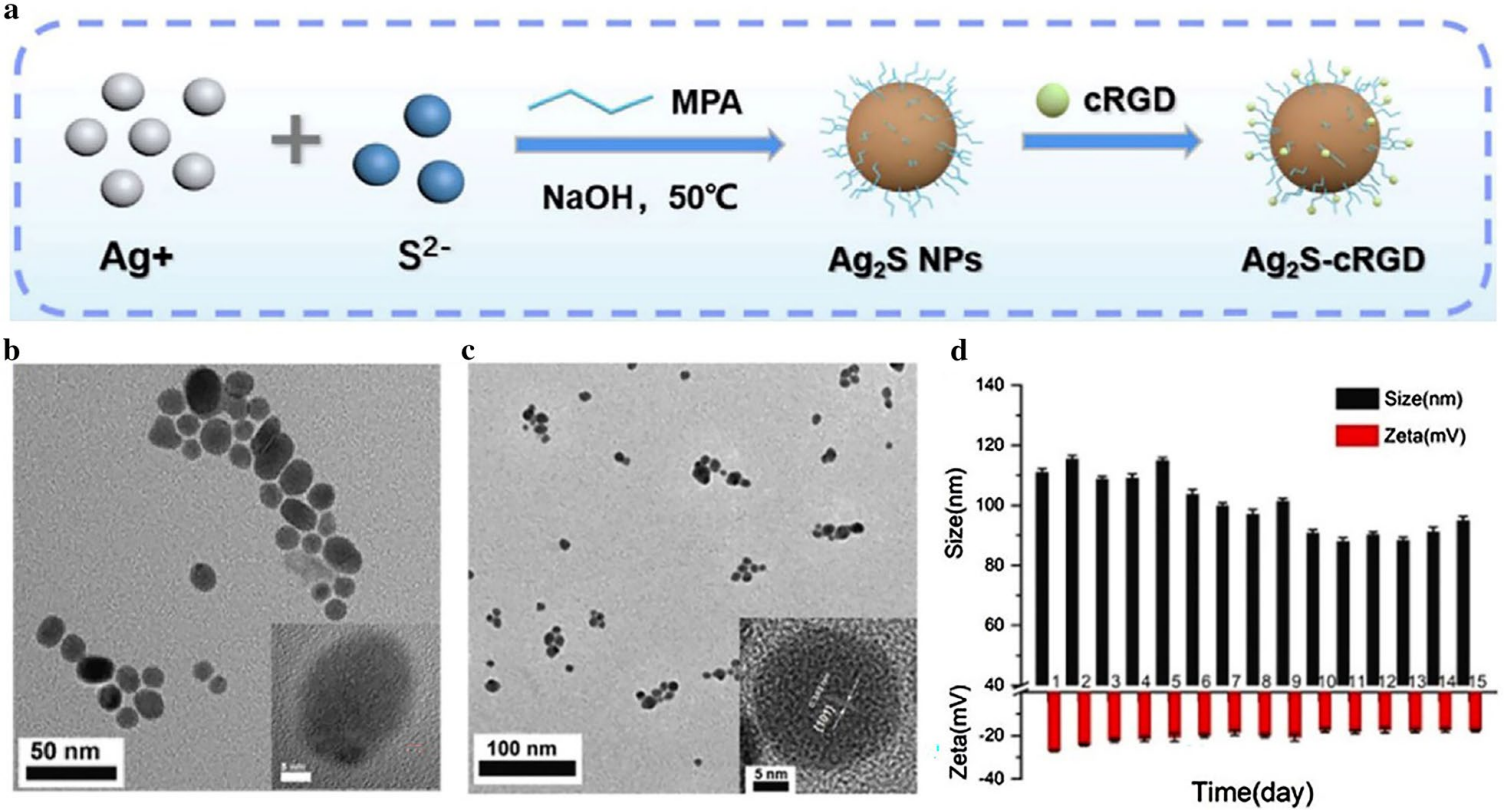
Schematic illustration presenting the fabrication process of Ag_2_S NPs by hydrothermal approach (**a**). TEM and high-resolution TEM image (inset) of Ag_2_S NPs (**b**) and Ag_2_S-cRGD NPs (**c**) with an Ag/S ratio of 2/1 at pH 6.0. Particle size and zeta potential stability of Ag_2_S-cRGD NPs for 15 days (**d**) (Reprinted with permission from Ref. [47]. Copyright 2020 Elsevier)

The hydrothermal approach can be applied to prepare nanoparticles such as CuS, silver sulfide (Ag_2_S), bismuth sulfide (Bi_2_S_3_), and molybdenum sulfide (MoS) by heating the dissolved metal salts and sulfide sources [44–47]. In the hydrothermal approach, metal chlorides or metal nitrates can be used as sources of metals, while sodium sulfides or organic sulfides can be used as sources of sulfides. This approach is easily accessible and inexpensive. The obtained nanomaterials possess good dispersion qualities [44, 45, 47]. Han et al. prepared highly dispersed Ag_2_S NPs through the hydrothermal approach (Fig. 2a) [47]. Researchers found that the morphology and particle size of Ag_2_S NPs could be controlled by adjusting the Ag/S ratios and the reaction pH. The transmission electron microscope (TEM) and high-resolution TEM image (inset) showed that Ag_2_S NPs and cyclic RGD modified Ag_2_S NPs (Ag_2_S-cRGD NPs) prepared with an Ag/S ratio of 2/1 at pH 6.0 had a particle size of about 15 nm (Fig. 2b, c). The as-obtained Ag_2_S-cRGD NPs had a monoclinic structure and good crystal structure with lattice fringes of 0.383 nm (Fig. 2c inset). Moreover, the results of the stability experiments showed that the dispersion stability of Ag_2_S-cRGD NPs was good in 15 days (Fig. 2d). However, the hydrothermal approach cannot be applied to moisture-sensitive ingredients (due to their poor stability, hydrolysis, and interactions in an aqueous environment), such as copper acetylacetonate and thioacetamide, or ingredients with low water solubility, such as sulfur powder and bismuth neodecanoate.

### Non-aqueous solvothermal approach

**Fig. 3:**
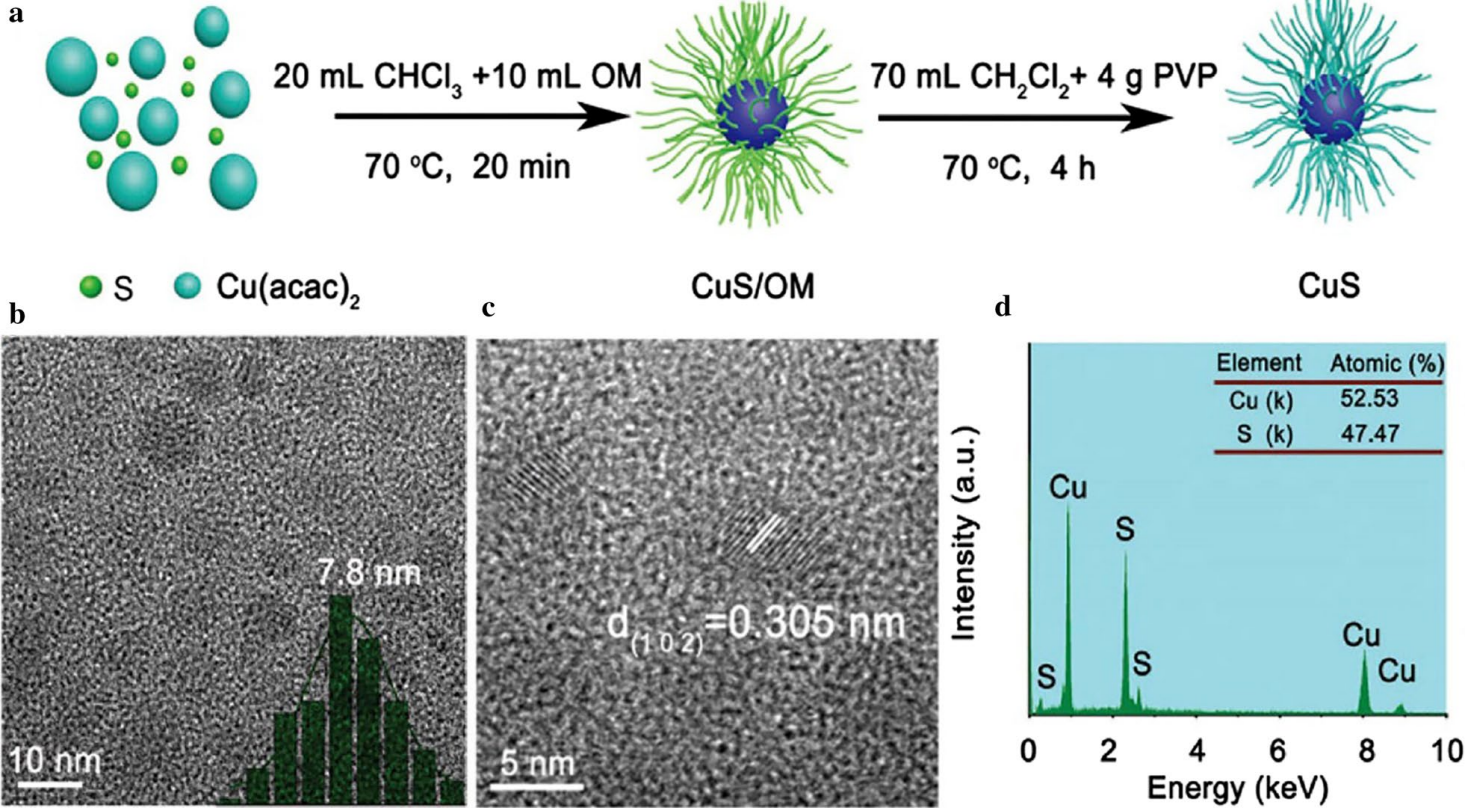
Schematic illustration presenting the fabrication process of CuS NCs by non-aqueous solvothermal approach (**a**). TEM image, size distribution, and high-resolution TEM image of CuS NCs (**b**, **c**). Energy-dispersive X-ray spectroscopy of CuS NCs (**d**) (Reprinted with permission from Ref. [52]. Copyright 2019 Royal Society of Chemistry)

The non-aqueous solvothermal approach, which was developed to overcome the defects of the hydrothermal approach, is a synthesis method using organic compounds and non-aqueous menstruum as solvents to react under a certain temperature and solution pressure. When applying the non-aqueous solvothermal approach, metal organics and organosulfur compounds are usually used as the metal source and sulfide source (Table 1) [48–51]. The CuS nanocrystals (CuS NCs) prepared by the non-aqueous solvothermal approach (Fig. 3a) were highly crystalline nanocrystals with a particle size of ~ 7.8 nm and a lattice spacing of ~ 0.305 nm (Fig. 3b, c) [52]. The as-prepared CuS NCs contained copper element and sulfur element, which were proved by energy-dispersive X-ray spectroscopy (Fig. 3d). In addition to CuS NCs, the non-aqueous solvothermal approach was well-established in the preparation of Bi_2_S_3_ NPs [49–51, 53]. The MeSNs prepared by this approach usually have uniform particle sizes and good dispersion qualities [48–50, 52]. However, the non-aqueous solvothermal approach also has certain flaws. This approach usually involves cumbersome synthesis procedures like using a high vacuum. Moreover, the obtained MeSNs by this approach may need complicated surface chemical modification to guarantee the hydrophilicity.

## Template‐assisted approach

**Fig. 4:**
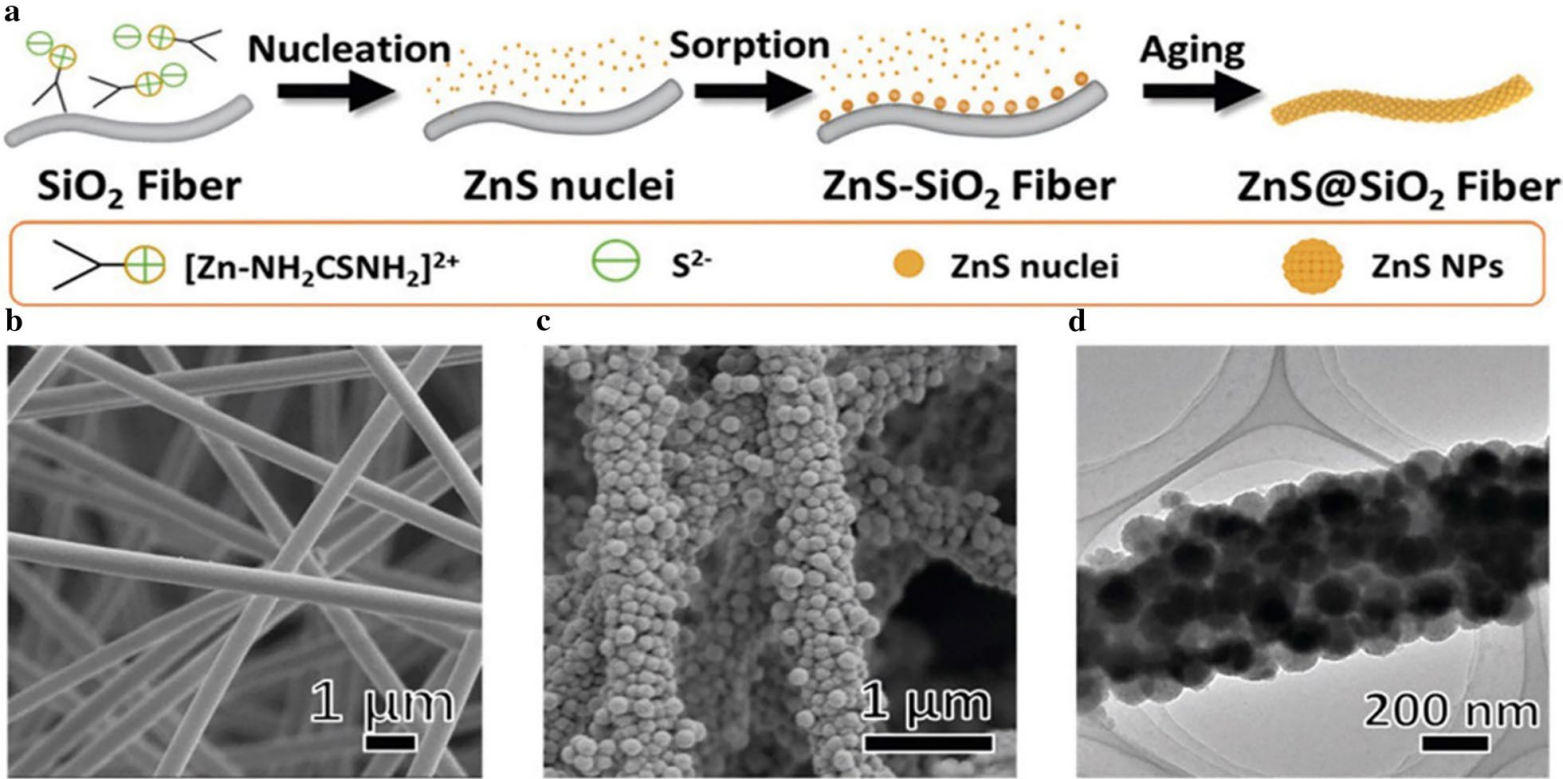
Schematic illustration showing the synthesis process of ZnS@SiO_2_ fibres by hard template-assisted approach (**a**). SEM image of as-spun SiO_2_ (**b**) and ZnS@SiO_2_ fibres (**c**). TEM image of ZnS@SiO_2_ fibres (**d)** (Reprinted with permission from Ref. [21]. Copyright 2020 Royal Society of Chemistry)

The template-assisted approach is the approach that uses porous nanosized material or colloidal dispersion as templates to precipitate MeSNs on their surfaces through adsorbing metal ions and/or sulfides. The ion adsorption caused by the templating agent accelerates the formation and mineralization of the seed. In the absence of the templating agents, 1.5–24 h is required to form nanosized metal sulfide [45–47]. While the time can be reduced to 15 min–2 h with the usage of a templating agent (Table 1) [12, 13, 19, 39]. The templates include hard templates (like mesoporous silica nanoparticles) and soft templates [such as cetyltrimethylammonium chloride (CTAC), PVP, or biopolymer melanin]. In the hard-templating approach, the particle size and morphology of MeSNs are highly correlated with the templating agent [21]. For instance, the silica fibre mesh (SiO_2_ nanofibres) was applied as hard templates for the fabrication of ZnS nanoparticle-decorated silica fibre mesh (ZnS@SiO_2_) (Fig. 4a, b). After the synthesis, ZnS nuclei formed in the precursor solution were adsorbed and grew on the surface of silica fibers [54], where ZnS NPs with an average diameter of ~ 110 nm were uniformly assembled (Fig. 4c, d).

**Fig. 5:**
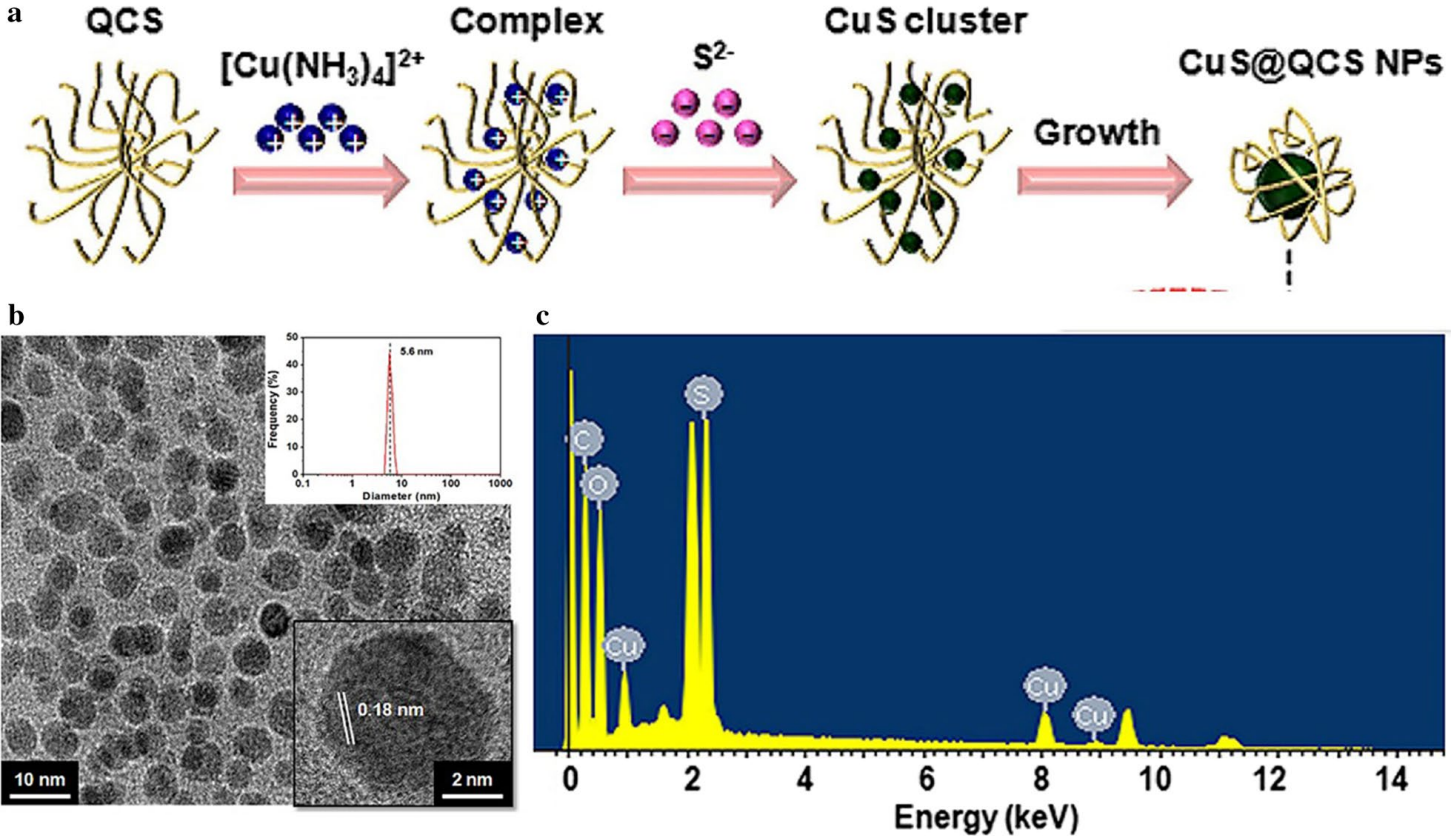
Schematic illustration showing the synthesis process of CuS@QCS-NPs by soft template-assisted approach (**a**). TEM image (**b**) and energy dispersive spectrometer (**c**) of CuS@QCS-NPs (Reprinted with permission from Ref. [19]. Copyright 2019 Elsevier)

The soft template usually does not have a defined geometry. It is self-assembled by amphiphilic copolymers which have threshold capacities in particular space areas, such as micro-emulsions, micelles, and biomacromolecules. Furthermore, these polymer materials can also be used as stabilizers to improve the dispersion and stability of MeSNs [52, 55]. As an example, the quaternized chitosan (QCS) was used as a soft biotemplate and stabilizing agent for the synthesis of QCS-template CuS composites (CuS@QCS-NPs) (Fig. 5a) [19]. Mechanically, the QCS molecules with numerous quaternary ammonium groups would form helical/coil chains that dispersed [Cu(NH_3_)_4_]^2+^ uniformly around the QCS molecules by electrostatic repulsion, providing potential nucleation sites for crystallization of CuS-NPs. With the introduction of Na_2_S, CuS nanoclusters were then formed in the [Cu(NH_3_)_4_]^2+^-enriched place by a metathesis reaction. And CuS nanoclusters were anchored on the positively charged QCS molecules, which acted as a template to direct the growing of CuS nanoclusters to nanoparticles. The as-prepared CuS@QCS-NPs were well-dispersed with a diameter around 5 nm (Fig. 5b) and were composed of Cu, S, C, and O elements (Fig. 5c). Although the soft-templating approach cannot prepare specific forms of MeSNs as the hard-templating approach, it can adjust the scale and structure of MeSNs by altering the type and concentration of templating agents. The template-assisted approach is the most flexible preparation approach as researchers can obtain MeSNs with specific structures and shapes through selecting different templates. Nevertheless, there are still some unavoidable problems encountered by the template-assisted approach. Firstly, soft templates (like CTAC) are toxic when incorporated in the synthetic process. Secondly, the synthesis method is relatively complex and the toxic components (such as organic solvent) may be introduced in the process of template removal. Finally, the large-scale production of MeSNs cannot be achieved via the template-assisted approach.

## Biomineralization approach

**Fig. 6:**
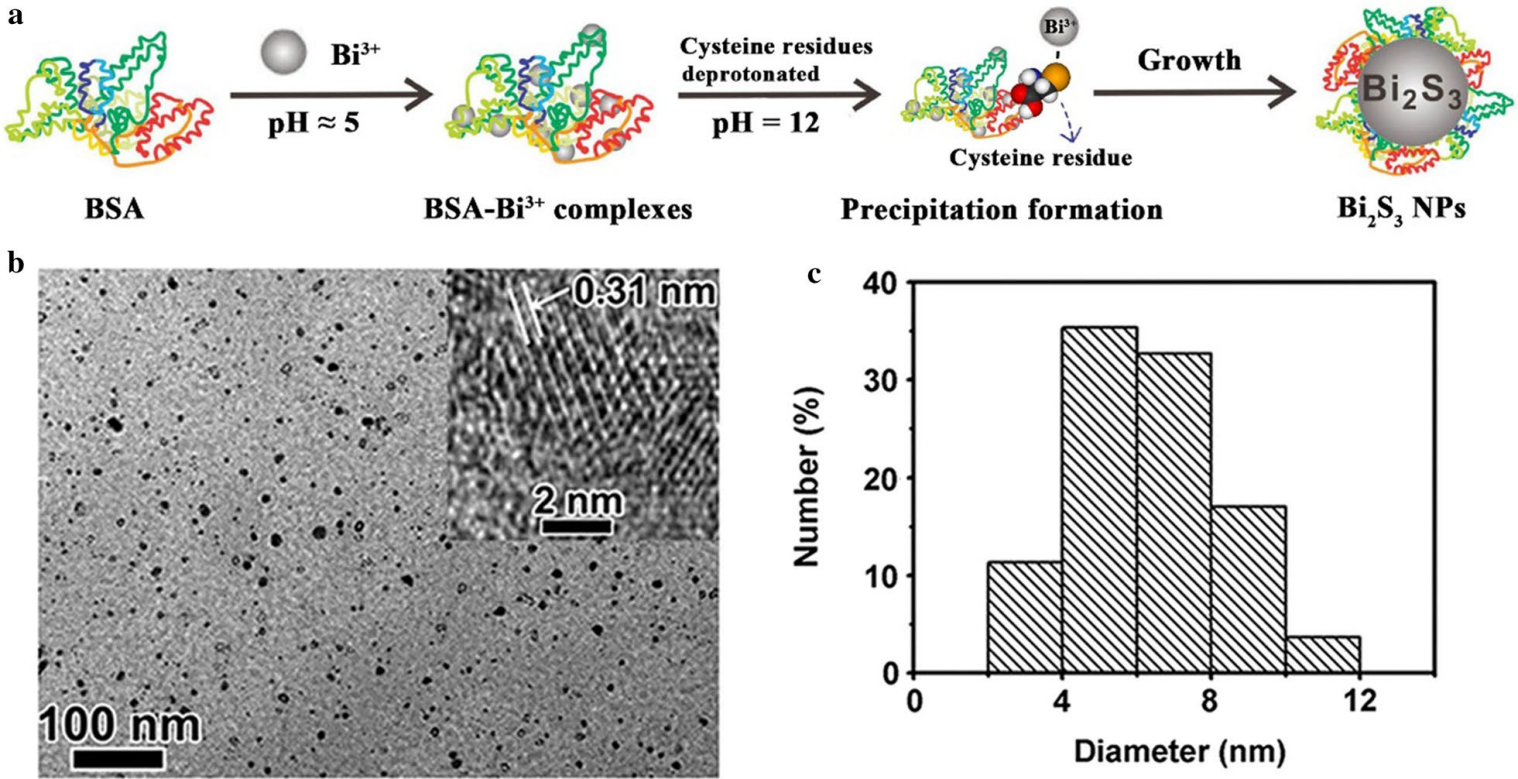
Schematic illustration of BSA-stabilized Bi_2_S_3_ NPs synthesized through a pH-mediated biomineralization approach (**a**). TEM image (**b**) and size distribution profile of Bi_2_S_3_ NPs (**c**) (Reprinted with permission from Ref. [31]. Copyright 2016 Wiley-VCH)

Biomineralization refers to the process of forming inorganic compounds via the biological regulation of biomacromolecules. During the biomineralization, metal ions will form coordination bonds with sulfur-containing groups of organic compounds and translate into solid minerals. No heating needed and using biological protein [such as bovine serum albumin (BSA)] as a sulfur source are the main differences between the biomineralization approach and the solvothermal approach. In the current fabrication approach, BSA not only serves as a sulfur precursor but also acts as a stabilizer for synthesizing the nanoparticles [15, 31, 56, 57]. Furthermore, the multifunctional groups of biomacromolecules also result in a variety of options for biofunctionalization on the surface [58–60]. As a typical case, the synthesis of Bi_2_S_3_ NPs comprises these steps (as displayed in Fig. 6a), i.e., (i) incubation of Bi(NO_3_)_3_ with BSA in an attempt to bind with Bi^3+^ ions via its functionalities (e.g., –COOH, –NH_2,_ and –SH) in the acidic media to form the BSA-Bi^3+^ complexes; (ii) following treatment with alkali, the complexes undergo degradation process to produce Bi_2_S_3_ NPs. BSA is known to undergo denaturation, thus releasing several residues (e.g. 35 cysteine residues) in alkaline conditions [58, 61], and cysteine is an outstanding source of sulfur for creating MeSNs [62, 63]. The majority of thiol groups within cysteine molecules are deprotonated under strongly basic conditions (pH ≈ 12) due to its pKa being 9.6 [59], which may increase the stabilization effect of BSA on the resulting Bi_2_S_3_ NPs. Therefore, a crucial role is played by the solution pH in forming BSA-stabilized Bi_2_S_3_ NPs. Wang et al. fabricated a kind of BSA-stabilized Bi_2_S_3_ NPs through the pH-mediated biomineralization approach [31]. The obtained nanomaterials were well-dispersed with a diameter of about 6.1 nm (Fig. 6b, c). The ultrasmall particle size of Bi_2_S_3_ NPs could be attributed to the strong multi-chelating feature of the BSA ligand.

To increase the efficiency of biomineralization, some researchers added Na_2_S or thioacetamide as the second sulfur source [14, 64–66]. In general, breeding BSA with metal ions drove the formation of metal-BSA complexes. Subsequently, the nucleation of MeSNs was sped up by bringing Na_2_S or thioacetamide into the mixture [65, 67]. In contrast to the solvothermal approach, the biomineralization approach is conducted at 37℃, which assures the immutability of BSA. Compared to other templating agents like silica nanoparticles and organic polymers, BSA exhibits higher biocompatibility with lower toxicity. Additionally, owing to its long blood circulation half time, albumin has manifested itself as an ideal carrier for drugs [35, 68]. In practice, BSA can be replaced with other bioactive proteins (such as whey protein, casein, collagen, and hemoglobin) or functional enzymes (such as lactate oxidase, glucose oxidase, and peroxidase) and the produced MeSNs will exhibit more powerful anti-tumor effect. However, MeSNs synthesized by the biological protein biomineralization approach can easily deteriorate. The requirements for post-processing (such as freeze-drying) and storage conditions of the product are relatively high. Moreover, this approach is still not suitable for mass production of MeSNs.

## Isomorphic substitution approach

**Fig. 7:**
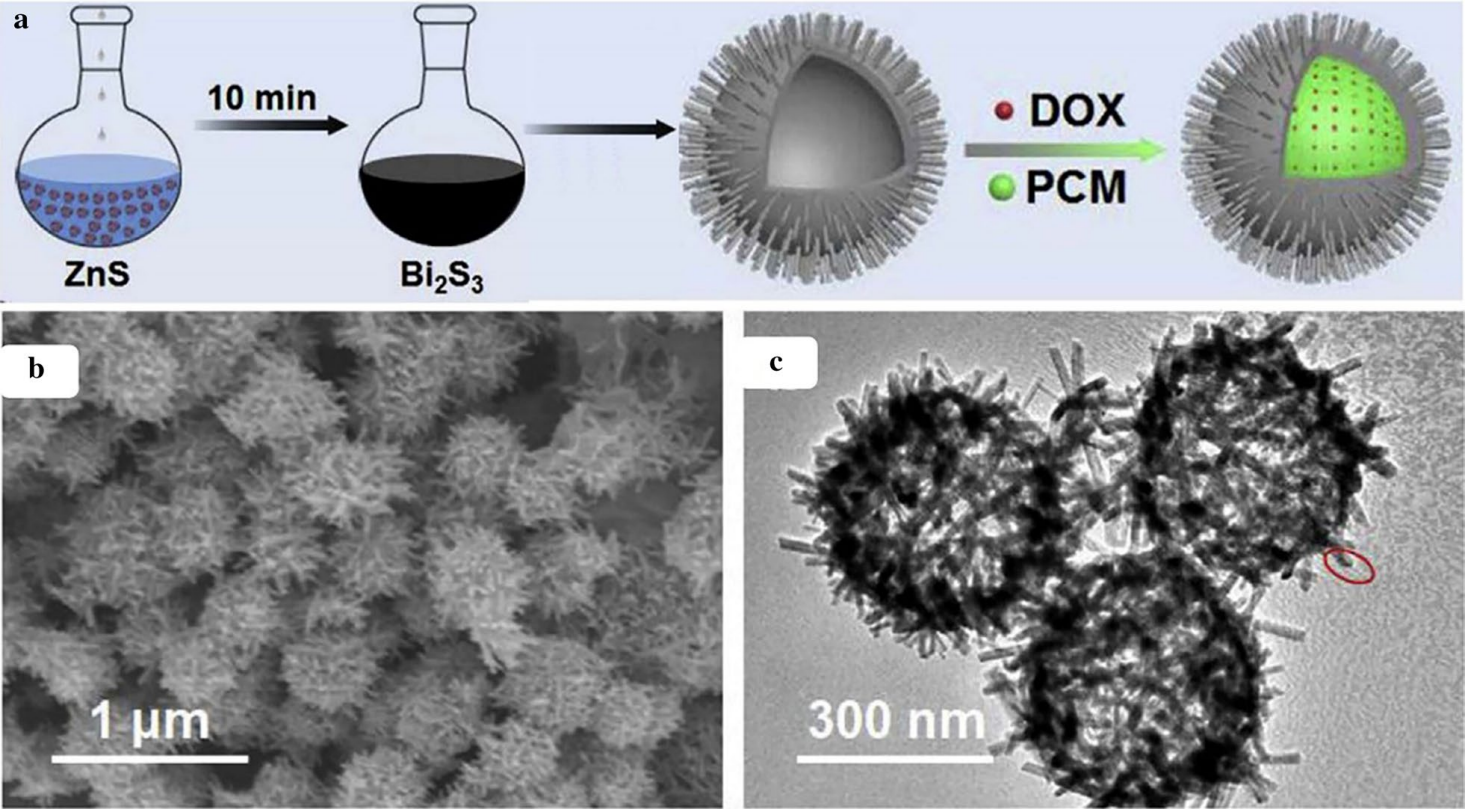
Schematic illustration presenting the fabrication process of drug loaded urchin-like Bi_2_S_3_ hollow microspheres by isomorphic substitution approach (**a**). SEM (**b**) and TEM (**c**) image of Bi_2_S_3_ hollow microspheres (Reprinted with permission from Ref. [69] Copyright 2020 Elsevier)

The isomorphic substitution approach is more complicated than the above approaches. It usually involves two steps. Firstly, the templates need to be synthesized [such as copper oxide (CuO) NPs or ZnS microspheres]. Then the metal ions or their ligands of the templates will be replaced in the solution via exchanging ions to form more stable MeSNs [18, 45, 69, 70]. Therefore, it is also named the ion exchange approach or sacrificial template approach [18]. For instance, Zhang et al. firstly synthesized ZnS composite microspheres. Then, bismuth nitrate ethylene glycol solution was added, and ZnS (Ksp = 2.5 × 10^− 22^, RT) composite microspheres would be transformed to highly insoluble Bi_2_S_3_ (Ksp = 1 × 10^− 97^, RT), which was thermodynamically favored because of ion exchange reaction (Fig. 7a) [69]. The scanning electron microscope (SEM) and TEM images showed that the Bi_2_S_3_ hollow microspheres were comprised of urchin-like hollow microspheres with an average diameter of about 280 nm (Fig. 7b, c). In the anion substitution approach, CuO NPs were firstly fabricated and then Na_2_S or (NH_4_)_2_ S was added in an alkaline environment. The sulfide replaced oxygen to form CuS NPs with lower solubility [45, 70]. Like the template-assisted approach, the morphology and size of the product can be controlled via the space region provided by the templating agent, which enables us to derive MeSNs with different structures [18, 69]. However, the purity of the synthesized MeSNs cannot be ensured. Moreover, the experimental process would be complicated compared with the template-assisted approach. Meanwhile, it is hard to achieve scale production by the isomorphic substitution approach.

## Liquid exfoliation approach

**Fig. 8:**
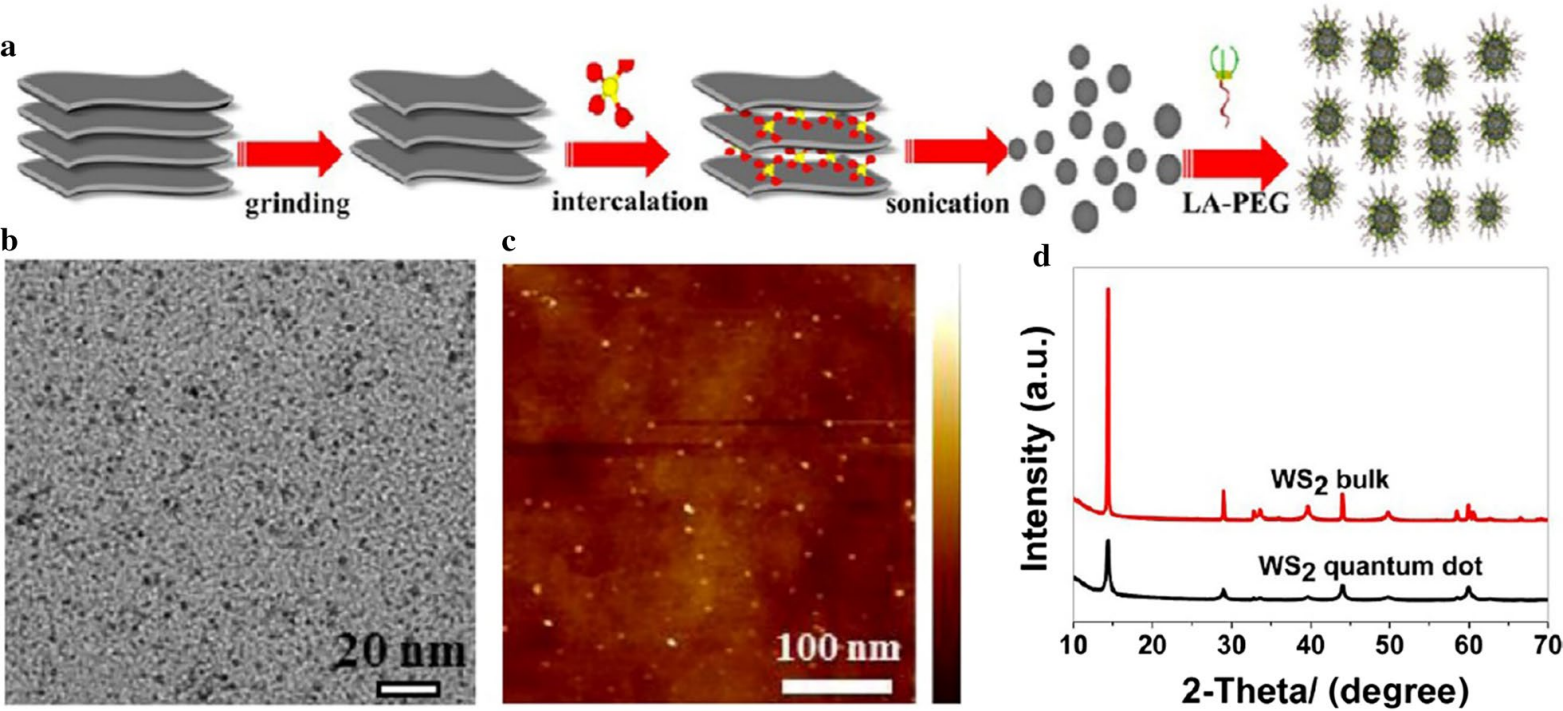
Schematic illustration presenting the fabrication process of WS_2_ quantum dots by liquid exfoliation approach (**a**). TEM image (**b**), atomic force microscopy image (**c**), and X-ray diffraction pattern (**d**) of WS_2_ quantum dots (Reprinted with permission from Ref. [33]. Copyright 2015 American Chemical Society)

The liquid exfoliation approach achieves industrial-scale production, which can be applied to the manufacturing of tin sulfide (SnS), tantalum sulfide (TaS_2_), or tungsten sulfide (WS_2_) nanomaterials. The ultrasound, microwave, shear stress, thermal stress, and electrochemistry are usually applied to remove or reduce the Van der Waals forces between layers of raw metal sulfides, so that nanoscale metal sulfides can be formed. Dispersing large-sized metal sulfides in an appropriate medium is a direct and efficient approach to reduce or eliminate Van der Waals forces. For example, SnS NSs and TaS_2_ can be exfoliated using *N*-methyl-2- pyrrolidone [40, 71, 72]. Concentrated H_2_SO_4_ can intercalate in layered WS_2_ to diminish Van der Waals forces and disperse the large-particle-size reactant into nanoscale particles with the assistance of ultrasonication (Fig. 8a) [33, 41]. The prepared WS_2_ quantum dots were 3 nm in diameter in TEM and atomic force microscopy image (Fig. 8b, c). The results of X-ray diffraction showed that all peaks corresponded to the characteristics of hexagonal WS_2_ (JCPDS card no. 08-0237) (Fig. 8d). However, this approach has a disadvantage of poor biocompatibility in medical application due to the high sedimentation rate of the prepared nanoparticles. To improve the biocompatibility of nanomaterials, surface modifications of MeSNs with PEG, lipid-PEG, or BSA were adopted by researchers [33, 40, 41]. Different from other approaches, the liquid exfoliation approach with physical and/or chemical effects can disperse the two-dimensional conversion metal sulfide material into nanosized particles. This preparation is relatively simple and efficient without the formation of metal-sulfur bonds. Thus, it is suitable for industrial production of MeSNs.

## Biosynthesis approach

**Fig. 9:**
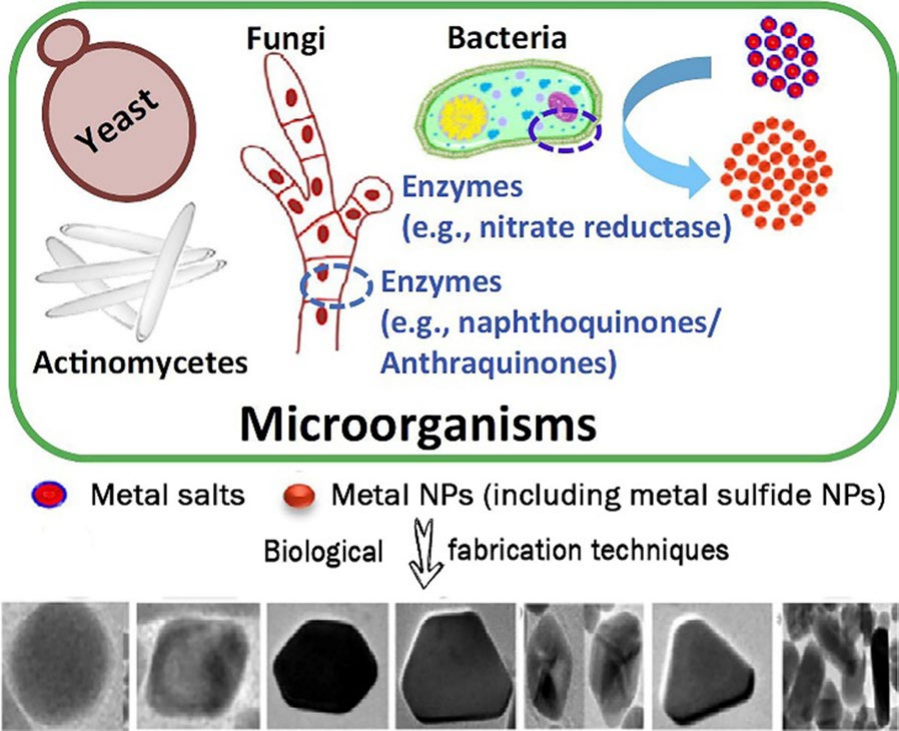
Biological synthesis of metal nanoparticles for biomedical applications (Reprinted with permission from Ref. [78]. Copyright 2016 Elsevier)

Although the above preparation approaches can prepare nanosized MeSNs, the high temperature, ultrasound, usage of surfactants, and highly explosive raw materials may cause safety issues and reduce the biocompatibility of products during the preparation [73]. Recently, the biomimetic approach was found to be an eco-friendly and safe alternative for preparing cadmium sulfide (CdS) NPs compared to conventional approaches [74, 75]. Several microorganisms, such as yeast, fungi, and algae, are available in the biosynthesis approach, which are energy conservation approaches without toxic chemicals, ultrasound, and high temperature (Fig. 9) [76–78]. Bacterial cells are recognized as valuable resources that have enormous potential as cost-effective, eco-friendly, and nontoxic replacements of traditional physiochemical procedures of synthesis. Bacteria possess the ability to accumulate and detoxify heavy metallic sources by making use of various reductase enzymes, thus leading to the reduction of cadmium salts to CdS particles with a nanosized distribution range [78]. The *Shewanella oneidensis* (*S. oneidensis*), which is a class of metal-reducing bacterium, is known for its special sulfate-reducing and anaerobic respiratory capacity [79, 80]. Hence, *S. oneidensis* bacterium can be used to study cadmium ions immobilization and anaerobic biofabrication of CdS NPs [81].

Colon cancer is a disease with high morbidity across the world [82]. Notably, a great amount of H_2_S is generated around the colon cancer cells due to the overexpression of cystathionine-β-synthase, which is a type of H_2_S generating enzyme. Our group reported a kind of paramagnetic iron oxide-hydroxide nanospindles (FeOOH NSs) for sensing and removing H_2_S gas [16]. Interestingly, FeOOH NSs could form nanosized FeS through ion exchange after the absorption of H_2_S. This novel approach used highly expressed pathological molecules as a sulfur source and synthesized ultra-small FeS NPs (about 5 nm) through ion exchange at the lesion site. At the same time, it could avoid the degradation of FeS NPs in the systemic circulation and thus reduce the side effects. Although biosynthesis has many advantages, the types of MeSNs prepared by this approach are limited. Furthermore, this method cannot achieve industrial-scale production.

## Other approaches

Apart from the commonly used approaches above, the mechanochemical approach and pyrolytic approach can also be applied to prepare MeSNs for medical application. Dash et al. reported the preparation of ZnS NPs by heating zinc monomeric complex ([Zn(SCN)_2_(2-benzoylpyridine)_2_]) at 620 °C for 2 h in a muffle furnace [23]. The obtained ZnS NPs were then washed with methanol and water to remove impurities. Another study reported that the ZnS nanocrystals could be synthesized through the sodium sulfide and zinc acetate precursors using a mechanochemical route in MiniCer, which is primarily a laboratory circulation mill [83]. The ZnS nanocrystalline sample was subjected to a wet milling process with a speed of 3500 rpm and a duration of 120 min in the presence of chitosan aqueous solution. After that, the obtained nanosuspension was centrifuged and stored at 4 °C for further usage.

## Choose the appropriate approach according to the usage purpose

Every preparation approach has its advantages and limitations. Researchers should choose the appropriate one according to their usage purposes. For example, the solvothermal approach and mechanochemical approach should not be used for the preparation of drug-loaded nanoformulations in one step, as the mechanical strength and molecular structure of drugs will be impaired by high temperature. Nanomaterials with special structures and morphology can be prepared through the template-assisted approach and isomorphic substitution approach. For instance, the use of hollow nanoparticles as templates to produce hollow MeSNs can improve the carrying capacity and assist the controlled release of the drug. The liquid exfoliation approach can be applied to achieve industrial-scale production. The microorganisms that reduce metal salts to MeSNs with a narrow size distribution are regarded as important nanofactories. They are eco-friendly and cost-effective, without toxic chemicals and high energy demand during the physiochemical synthesis.

## Application in cancer therapy

All the discussed preparation approaches are aimed to obtain bioactive MeSNs with good stability and high biosafety for medical applications. After summarizing the previous studies, the role of MeSNs in cancer therapy can be classified into six categories: (i) MeSNs with special structure and composition can be used as carriers of anti-tumor drugs; (ii) the MeSNs with high light absorption coefficient, such as CuS, WS_2_, MoS_2_, and vanadium sulfide (VSx), can be used as phototherapeutic agents; (iii) high atomic number metal-containing MeSNs (such as WS_2_ and Bi_2_S_3_) can be applied for radiotherapy; (iv) MeSNs that will degrade in the acidic tumor microenvironment (TME) can be used as gas-generating agents (such as FeS and MnS); (v) and the released Fe^2+^ and Mn^2+^ can act as Fenton catalysts for chemodynamic therapy (CDT); (vi) MeSNs that can stimulate immune responses in the body can be used as adjuvants to participate in immunotherapy. This section will classify and introduce the roles of MeSNs in cancer therapy deeply (Table 2).

**Table 2:** The classification of functional MeSNs for cancer therapy

Therapy strategies	Functional MeSNs	Cargoes	Release patent	Synergistic treatment	Ref.
Photothermal therapy	Chitosan-stabilized CuS NPs	NA	NA	NA	[19]
	RGD and TAT peptides modified mesoporous silica coated CuS NPs	NA	NA	NA	[12]
	Clearablemanganese-doped CuS nanodots	NA	NA	NA	[144]
	Ni_9_S_8_ NPs	NA	NA	NA	[45]
	Bi_2_S_3_-gold heterojunction nanorods	NA	NA	NA	[51]
	Nanoceria decorated flower-like MoS_2_ nanoflakes	NA	NA	NA	[46]
	Gold/gold Sulfide NPs	NA	NA	NA	[146]
	ReS_2_ NPs	NA	NA	NA	[22]
	BSA and PEG modified RuS_1.7_ nanoclusters	NA	NA	NA	[98]
	Cyclic RGD modified Ag_2_S NPs	NA	NA	NA	[47]
	Erythrocyte-cancer hybrid membrane camouflaged hollow CuS NPs	DOX	Photothermal sensitive	Chemotherapy	[39]
	Doxorubicin and chlorin e6 loaded hollow CuS NPs	Dox and Chlorin e6	Photothermal sensitive	Chemotherapy	[85]
	Mesoporous SiO_2_ encapsulated CuS NPs	siRNA and Adriamycin	Photothermal sensitive	Chemotherapy	[90]
	Hollow mesoporous NiS NPs	DOX	pH sensitive	Chemotherapy	[48]
	Antibody-functionalized Bi_2_S_3_@mesoporous silica core-shell NPs	DOX	pH and temperature sensitive	Chemotherapy	[89]
	SnS nanosheets	DOX	NA	Chemotherapy	[72]
	Polyethylene glycol TaS_2_ nanosheets	DOX	Photothermal and moderate acidic pH sensitive	Chemotherapy	[40]
	Urchin-like Bi_2_S_3_ NPs	DOX	Photothermal sensitive	Chemotherapy	[69]
	PEG modified iron oxide-hydroxide nanospindles	NA	pH sensitive Fe^2+^ release	PTT	[16]
	WS_2_ nanosheets	Photosensitizer	NA	PDT	[41]
Photodynamic therapy	Co_9_S_8_ nanodots	NA	NA	PTT	[67]
	Ag_2_S NPs	NA	NA	NA	[66]
	Transferrin modified hollow mesoporous CuS NPs	Artesunate	pH sensitive	Chemotherapy	[70]
	Bi_2_S_3_ nanorods	Zinc protoporphyrin	NA	NA	[50]
Radiotherapy	Melanin-PEG coated CuS NPs	DOX	pH sensitive	Chemotherapy	[13]
	Bi_2_S_3_@BSA	MTX	Proteinase	Chemotherapy	[57]
	Folic acid conjugated Bi_2_S_3_@BSA	Curcumin	Sustained release	Chemotherapy	[56]
	Platelet membrane camouflaged mesoporous silica-coated Bi_2_S_3_ nanorods	NA	NA	PTT	[49]
	Ultrasmall Bi_2_S_3_ NPs	NA	NA	PTT	[31]
	Gold-gold sulfide nanoshells	NA	NA	NA	[55]
	Lipoic acid-PEG modified WS_2_ quantum dots	NA	NA	PTT	[33]
Chemodynamic therapy	Layered double hydroxides-CuS nanocomposites	NA	Photothermal active Cu^2+^ release	PTT and PDT	[30]
	PEG modified iron oxide-hydroxide nanospindles	NA	pH sensitive Fe^2+^ release	PTT	[16]
	PVP-modified CuS nanocrystals	NA	Photothermal active Cu^2+^ release	PTT and PDT	[52]
Gas therapy	Ferrous sulfide embedded FeS@BSA nanoclusters	NA	pH sensitive H_2_S release	CDT	[14]
	MnS NPs	NA	NA	CDT	[64]
	PVP-modified multifunctional Fe_1 − x_S NPs	NA	pH sensitive H_2_S release	PTT and CDT	[140]
	ZnS NPs-decorated silica fibre mesh	DOX	pH sensitive H_2_S release	Chemotherapy	[21]
Immunotherapy	Maleimide polyethylene glycol modified CuS NPs	Tumor antigens	Photothermal sensitive	PTT	[44]
	Bi_2_S_3_ NPs	Ganoderma lucidum polysaccharide	NA	Radiotherapy	[15]

## Drug delivery

Chemotherapy is a frequently used approach in cancer therapy that prevents the metastasis and recurrence of tumors. Although chemotherapeutic drugs have achieved great strides in medical science, short half-life, poor solubility, nonspecific distribution, fast clearance, and narrow therapeutic index are typical factors that limit their applications because of extensive systemic toxicity [84]. To address the pharmacological challenges, Yang et al. engineered polydopamine (PDA) coated hollow mesoporous nickel sulfide (NiS) NPs (hm-NiS) for the delivery of doxorubicin (DOX) [48]. The encapsulation efficiency and loading capacity of DOX were respectively estimated as 66.9 and 7.1%. The high efficiency of drug encapsulation not only resulted from the internal cavity and hollow mesoporous structural framework of hm-NiS but also from the strong interaction between DOX and PDA. Importantly, both the acidic environment of tumor tissue and NIR laser irradiation led to the stimulus-responsive drug release due to the protonation of -NH_2_ groups in the DOX molecules and local thermal shock, respectively. When exposed to NIR laser irradiation, the designed drug delivery nanosystems had a promising tumor growth inhibition index of 91.8% after 14 days post-treatment on 4T1 tumor-bearing mice. In another study, Li et al. designed a kind of mesoporous hollow CuS nanoparticles (H-CuS NPs) for delivering chlorin e6 (Ce6, a kind of photosensitizer) and DOX to tumor sites [85]. The thermo-responsive degradation feature of H-CuS NPs could trap drugs interiorly in the cavity of H-CuS nano vehicles, thus functioning as a removable plug and thereby attained the controlled release of drugs by light-induced thermal stimuli. The attribute was highly useful for targeted delivery of the drug, which only caused a minimal release of drug nonspecifically in the circulation, thereby enhancing the drug bioavailability in tumor tissues via improved permeability and retention effects. With the assistance of laser irradiation, the tumor volume after applying the prepared nanodrugs gradually decreased to almost 20% of its original size with a tumor growth inhibition index of 98.4%.

In addition to the hollow MeSNs prepared by the template-assisted approach, the MeSNs with layered nan-structure also have ideal performance in the field of drug loading and delivery [86–88]. Notably, Xie et al. engineered a kind of two-dimensional tin sulfide nanosheets (SnS NSs) with a high loading rate of DOX (up to about 200% in weight) through electrostatic absorption between the negative potential carriers and positively charged DOX [72], which was larger than that of mesoporous MeSNs (about 7%) [48]. A sheet structure with layers furnishes an extensive surface area for efficiently loading drugs via numerous intermolecular interactions including Van der Waals forces, π-π stacking, hydrophobic interactions, and electrostatic forces. Efficient drug delivery capabilities make MeSNs more attractive drug carriers in the field of tumor treatment. Although most of MeSNs are solid without internal spaces for loading drugs, these nanomaterials can still achieve efficient delivery and control the release of small molecules (such as DOX or siRNA) after coating with mesoporous silica or organic polymers shell [89, 90].

## Phototherapy

Researchers have shown a rising interest in the realm of cancer therapy while seeking numerous advantages like better controllability, negligible invasiveness, high efficacy, and selectivity [91]. In comparison to conventional radiotherapy and chemotherapy, the selective treatments involving phototherapies lower the systemic toxicity and drug resistance significantly [92, 93]. Among various light-sensitive materials, bioactive MeSNs are recognized as prospective core materials owing to their excellent properties, such as extraordinary NIR optical absorption, high molar extinction coefficient, metabolizability by humans, and high photothermal conversion efficiency [12]. During the past decade, researches have shown that MeSNs-based nanotherapeutics with light-absorbing ability can transfer energy to surrounding oxygen to generate highly active singlet oxygen (^1^O_2_) or transform it into heat energy. The MeSNs-based phototherapy can be further classified into photothermal therapy and photodynamic therapy.

### Photothermal therapy

In photothermal therapy (PTT), light-harvesting agents give rise to heat under the influence of light irradiation, leading to the thermal ablation of cancer tissues. Upon absorbing light of a specific wavelength range, vibrational energy is transformed by the activated light-harvesting agents or materials into heat energy as their electrons/atoms return to the ground state [94, 95]. A relaxation process that is non-radiative like this will result in rapid local transformation of light into heat, which can increase the temperature of tumor sites sufficiently to eradicate tumor cells. A high NIR light absorbance is manifested by ideal light-harvesting nanoagents because of their in-depth penetration within tissues and thus they possess an efficient photothermal effect [96]. To reduce the side effects of the material itself, the agents or materials for PTT should be biocompatible and nontoxic [97].

**Fig. 10:**
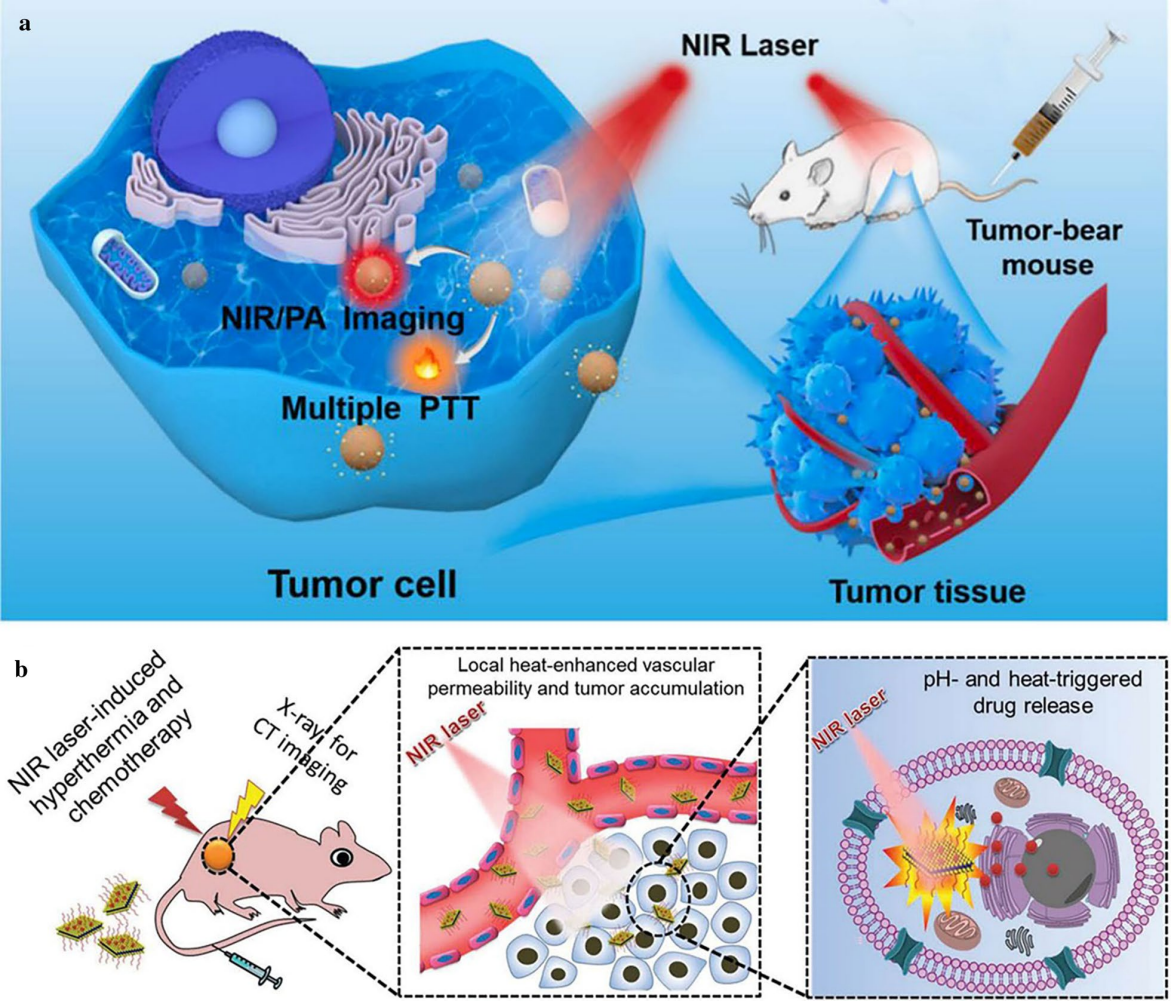
Schematic illustration showing the application of theranostic Ag_2_S NPs for PTT (**a**). Reprinted with permission from ref [47]. Copyright 2020 Elsevier. Schematic illustration presenting the multifunctions of PEG-TaS_2_-DOX against tumor (**b**) (Reprinted with permission from Ref. [40]. Copyright 2017 Wiley-VCH)

Compared with other photosensitive nanomaterials like carbon and gold-based nanocrystals, the benefits of MeSNs are their low cost, easy fabrication, biodegradability, and rapid metabolism. MeSNs can convert optical energy into thermal energy to kill tumor cells by triggering necrosis mediated mechanisms and/or disrupting the cellular structure. For example, the cyclic RGD peptide modified Ag_2_S NPs (Ag_2_S-cRGD) with optimal particle size (~ 15 nm) were synthesized and used as PTT agents [47]. During the photothermal conversion, the obtained Ag_2_S-cRGD composites manifested strong NIR absorbance, and tumors were effectively suppressed or even eliminated without metastasis or recurrence after two or three photothermal treatments (Fig. 10a). Photothermal transformation is the most common property of MeSNs. Various kinds of MeSNs had been demonstrated as excellent PTT-based nanoplatforms for cancer therapy, such as NiS NPs [45], molybdenum sulfide nanoflakes [46], rhenium sulfide NPs [22], tantalum sulfide (TaS_2_) nanosheets (NSs) [40], and ruthenium sulfide nanoclusters [98]. For instance, a novel tantalum-based multifunctional nanoplatform composed of biocompatible TaS_2_ NSs and DOX (PEG-TaS_2_-DOX) was designed for simultaneous PTT [40] (Fig. 10b). The in vivo antitumor study showed that the tumor temperature in the PBS group was increased by < 4 °C after NIR irradiation, whereas the tumor temperature in the PEG-TaS_2_ or PEG-TaS_2_-DOX group was increased by about 24–26 °C to over 60 °C after the same irradiation period. As a result, the tumor growth was remarkably suppressed in the PEG-TaS_2_-treated group with laser irradiation, and more impressively, tumors were eliminated in the mice treated with PEG-TaS_2_-DOX followed by laser irradiation without causing significant weight loss. The PEG-TaS_2_ NS platform was expected to create a new way for developing more effective PTT-based therapeutic agents for cancer therapy.

Highly expressed H_2_S is the most representative pathological feature of colon cancer [82]. Within colon cancer cells, notably, a great amount of H_2_S is generated due to H_2_S-producing cystathionine-β-synthase overexpression. The endogenous H_2_S produced can promote the proliferation of colon cancer cells and angiogenesis around the tumor tissue [99–101]. For sensing and removing H_2_S gas, our group reported a kind of paramagnetic iron oxide-hydroxide nanospindles (FeOOH NSs) with high adsorption capacity and reactivity of H_2_S at ambient pressure and room temperature [16]. Importantly, FeOOH NSs would form nanosized FeS through ion exchange after the absorption of H_2_S. The produced FeS NPs had a high photothermal conversion capability which targeted the tumor sites. The multifunctional FeOOH NSs exhibited powerful PTT-assisted anticancer effects on colon cancer and held great potential for future clinical translation. Significantly, the treatment strategy proposed in our research may promote a new trend in endogenous H_2_S-derived disease therapy.

### Photodynamic therapy

The central idea behind photodynamic therapy (PDT) is the accumulation of oxygen, nontoxic photosensitizers, and the generation of cytotoxic reactive oxygen species (ROS) from light, such as ^1^O_2_, to destroy target cells or tissues selectively [102]. In the generating process of ^1^O_2_, the singlet state of the photosensitizers or photo-sensitive nanomaterials will be produced under the irradiation of light with an appropriate wavelength range. These singlet materials will undergo the process of intersystem crossing to form the triplet state, which could transfer the energy to the triplet state of oxygen and generate ^1^O_2_ [103]. Similar to PTT, PDT also manifests obvious benefits, including negligible invasiveness, better controllability, decreased side effects, as well as high efficacy and selectivity in tumor treatment [104].

**Fig. 11:**
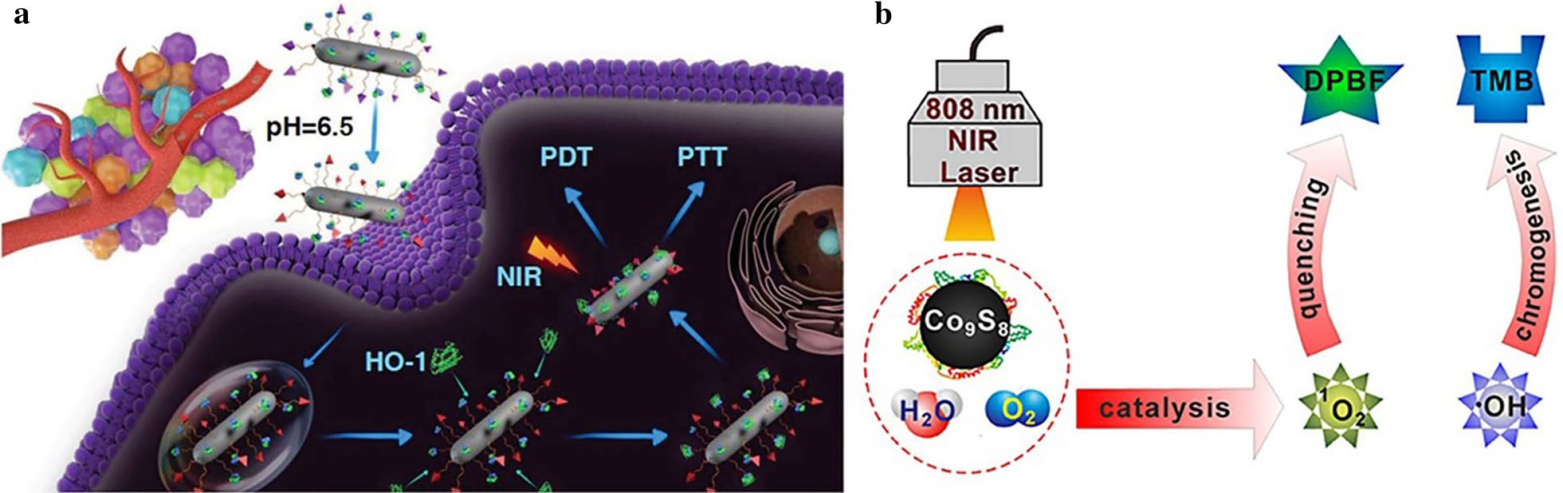
Illustration of the phototherapeutic effect of BPZP nanosystems (**a**). Reprinted with permission from ref [50]. Copyright 2019 Wiley-VCH. Schematic illustration of the photocatalytic activity of Co_9_S_8_ NDs upon NIR irradiation (**b**) (Reprinted with permission from Ref. [67]. Copyright 2018 American Chemical Society)

Among various kinds of MeSNs, NIR laser can be used to excite Bi_2_S_3_ to generate free holes in the valence band and electrons in the conduction band, which can react with water and oxygen to form hydroxyl and superoxide radicals respectively for potential NIR-activated PDT. On the basis of this feature, Cheng et al. constructed smart Bi_2_S_3_ nanorods (Bi_2_S_3_ NRs) with a potent photodynamic property (Fig. 11a) [50]. Zinc protoporphyrin IX (ZP) was associated with Bi_2_S_3_ NRs through a thermo-responsive polymer to prepare Bi_2_S_3_ NR-P(NIPAM-*co*-AM)-ZP-Pep nanosystems (BPZP). Under the irradiation of NIR laser, the bending of the band at the interface of Bi_2_S_3_ and ZP induced a built-in field. The energy edge of the highest occupied molecular orbital in ZP was higher than that of the valence band in Bi_2_S_3_ NRs. Thus, it led to the transformation of NIR laser-triggered holes from Bi_2_S_3_ to ZP. This phenomenon promoted efficient electron-hole spatial separation and subsequent ROS production as a result of the PDT effect. Therefore, BPZP exhibited the most pronounced tumor growth inhibitory effect with a tumor growth inhibition rate of 95.3% under NIR laser irradiation.

In another study, Lin et al. reported a kind of enzyme-like cobalt sulfide nanodots (Co_9_S_8_ NDs) for photodynamic cancer therapy [67]. The constructed peroxidase-like Co_9_S_8_ NDs not only possessed near-infrared absorption ability but also generated ROS (·OH and ^1^O_2_) via photocatalytic reaction (Fig. 11b). Furthermore, NIR light could improve the peroxidase-like activity of Co_9_S_8_ NDs and increase the efficiency of ROS production. Under NIR irradiation, Co_9_S_8_ NDs completely suppressed the tumor growth and even eradicated the tumor. In general, MeSNs-mediated PDT is effective in the treatment of tumors.

## Radiotherapy

As one of the most common and effective treatments of tumors, radiotherapy (RT) makes use of X-rays/γ-rays or other high-energy ionizing radiations to kill tumor cells via direct interaction with biomolecules or indirect radiolysis of water molecules within tumor cells to create free radicals and thus cause oxidative damage [105, 106]. RT alone or in combination with other treatments such as chemotherapy and/or surgery is often used to treat most patients with malignant tumors [107, 108]. The dramatic advances of radiosensitizers further decrease the severe side effects of RT under high-dose ionizing radiation [109]. Materials with high Z (atomic number) like heavy metals are chemically inert with decreased risk of cellular toxicity, thus can be used in the clinic [110]. Over the past decade, a number of studies focused on radiosensitizing effects of metal-based NPs for RT have been reported [111]. Auger electrons and photoelectrons coming from the metal-based irradiated NPs can enhance the dose of radiation particle beam and subsequently lead to radiobiological improvement [112, 113]. The high atomic number and X-ray attenuation coefficient of Bi (Z = 83) make Bi-based NPs suitable for X-ray radiosensitive and cancer diagnostic therapeutic agents [114, 115]. Nosrati et al. fabricated a kind of Bi_2_S_3_ NPs as biocompatible and targeted nano-radiosensitizers to be employed as carriers of curcumin (Bi_2_S_3_@BSA-FA-CUR) [56]. According to the result of in vivo X-ray RT, upon treatment with radiation and Bi_2_S_3_@BSA-FA-CUR, the mice tumors vanished in nearly three weeks. The effect of Bi_2_S_3_-based nanodrugs on radiosensitization had been further confirmed by other researches [13, 15].

**Fig. 12:**
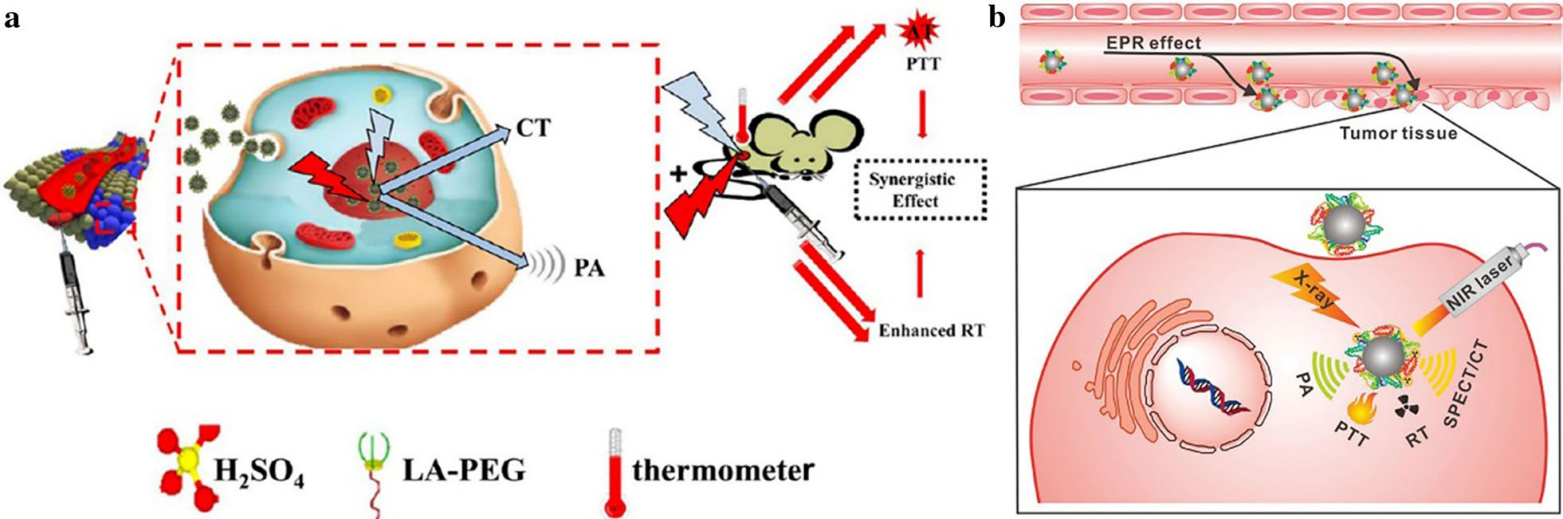
Schematic illustration of WS_2_ quantum dots for RT/PTT synergistic therapy (**a**). Reprinted with permission from ref [33]. Copyright 2015 American Chemical Society. Schematic illustration showing multiple theranostic functions of Bi_2_S_3_ NPs for PA/CT imaging, SPECT imaging upon radiolabelling, and RT/PTT synergistic therapy (**b**) (Reprinted with permission from Ref. [31]. Copyright 2016 Wiley-VCH)

Although metal-containing nanosystems-mediated RT has achieved great progress, many research reports suggested that it only had minimal efficiency in killing the hypoxic cancer cells [41, 116]. This is one of the major reasons for RT failure in the clinic. Fortunately, PTT can overcome the deficiency of hypoxia in RT-related treatment. The intratumoral blood flow can be increased by an appropriate level of hyperthermia and subsequently enhance the tumor oxygenation status, which may result in cells becoming more sensitive to RT [117, 118]. To achieve PTT/RT synergistic therapy, Yong et al. constructed multifunctional tungsten sulfide (WS_2_) quantum dots as the radiosensitizer and photosensitizer, due to their high Z number and NIR absorbing feature (Fig. 12a) [33]. Upon irradiation with 808 nm laser, the prepared WS_2_ quantum dots which had a 3 nm average diameter not only produced significant heat but also simultaneously generated dose-enhancement effects of RT. Compared to RT alone (tumor growth inhibition index = 37.64%), the integration of WS_2_ quantum dots and RT (tumor growth inhibition index = 56.85%) showed more effective inhibition on tumor growth, indicating the efficient sensitization effect of WS_2_ quantum dots on RT. In another study, Wang et al. constructed a kind of 10 nm Bi_2_S_3_ biocompatible particles for PTT/RT synergistic cancer therapy (Fig. 12b) [31]. Due to the remarkable photothermal conversion efficiency and large X-ray attenuation coefficient, the implanted tumors were completely eradicated through combined therapies. The synergistic effect might contribute to the above phenomena, of which Bi_2_S_3_ NPs-mediated RT killed the radiosensitive cells deep inside the body while PPT damaged the radio-resistant hypoxic cells and superficial cancer cells [119]. Mice treated with saline or Bi_2_S_3_ NPs alone were dead around 30 d post-treatment, owing to the malignant proliferation and abnormal lung metastasis of the tumor. However, mice that received combined treatment of PTT and RT after intravenous injection of the Bi_2_S_3_ NPs presented a survival rate of 100% over 40 d post-treatment. All these studies implied that the RT-assisted synergistic treatment strategies held great potential in tumor suppression.

## Chemodynamic therapy

Chemodynamic therapy (CDT), which promotes the destruction of tumor cells or increases their susceptibility to other tumor treating strategies such as RT, chemotherapy, and phototherapies, is an emerging therapeutic technique. CDT majorly works by amplifying intracellular oxidative stress [120, 121]. CDT triggered by intracellular Fenton or Fenton-like reactions can destruct tumor vasculature, damage plasma membranes and DNA, and stimulate an antitumor immune response, thereby mediating the antitumor activity by apoptosis and/or other cell death pathways [122]. High hydrogen peroxide (H_2_O_2_) levels ranging from 1 mM to 100 mM are often present within tumor cells due to abnormal metabolic processes, rendering this approach viable [123]. The so-called Fenton reaction is one of the major CDT strategies, involving the reaction between transition metal (Fe^2+^, Cu^2+^, or Mn^2+^) and endogenous H_2_O_2_ to generate hypertoxic hydroxyl radicals (·OH) in tumor areas [14, 52, 64]. Based on this insight, various metal-containing nanomaterials were developed for ROS generated CDT during the past ten years [124]. Transition metal sulfide nanomaterials, which release metal ions after specific degradation in the acidic TME can upregulate the intracellular ROS through Fenton reaction or Fenton-like reaction. In this section, the anti-tumor mechanisms of MeSNs-related degradation products will be discussed in detail.

**Fig. 13:**
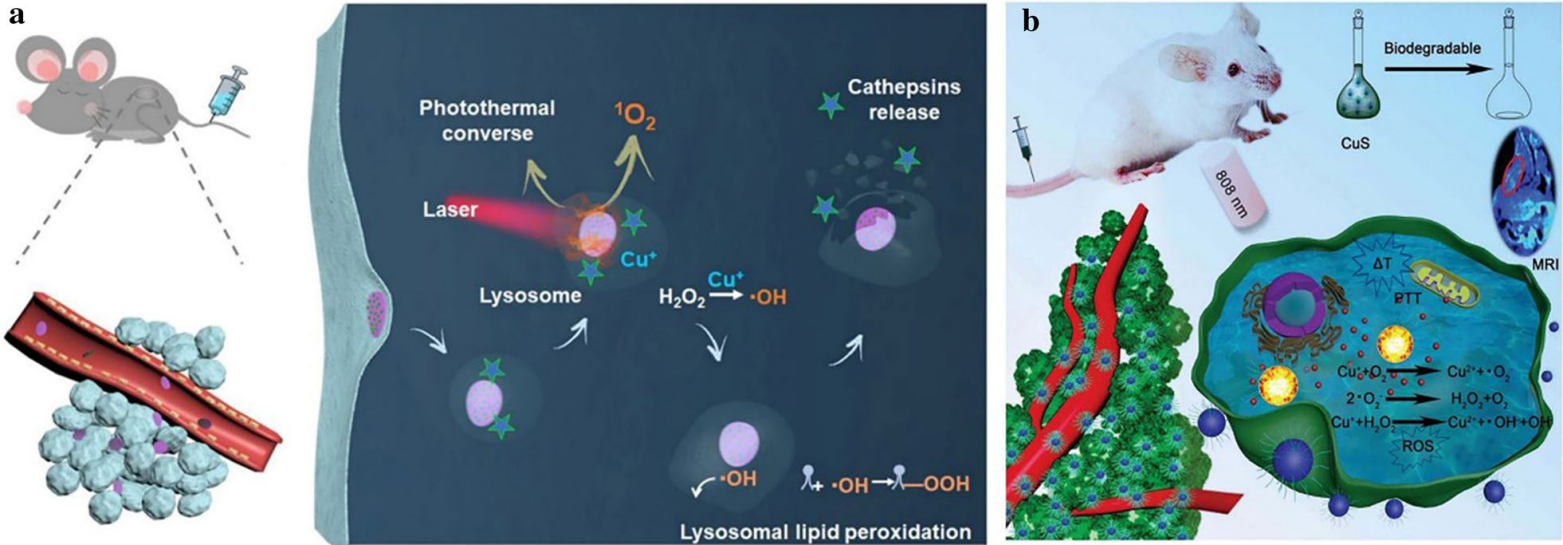
Schematic illustrating NIR-triggered cancer lysosome pathway death-assisted by LDH-CuS NCs (**a**). Reprinted with permission from ref [30]. Copyright 2020 Wiley-VCH. Schematic illustration of biodegradable CuS NCs for MRI-guided cancer therapy (**b**) (Reprinted with permission from Ref. [52]. Copyright 2019 Royal Society of Chemistry)

Cu^2+^ is a transition metal ion found in living bodies, which primarily exists in protein-bound forms and serves as a cofactor in a multitude of enzyme-catalyzed redox reactions. Cu^+^/Cu^2+^ redox couples have been reported to catalyze Fenton-like reactions efficiently under weakly acidic or neutral conditions with a reaction rate of 1 × 10^4^ M^− 1^ s^− 1^, which is up to 160-fold greater than that of Fe^2+^ [125, 126]. According to the above mechanistic details, a biodegradable platform was designed by Liu et al. based on the layered double hydroxide-copper sulfide nanocomposite (LDH-CuS NCs) (Fig. 13a) [30]. It has been reported that CuS nanodots would degrade under NIR light [52]. The exposure to laser light increased the temperature of LDH-CuS NCs sites, which reduced the stability of LDH-CuS NCs when encountered with local temperature differences and thus induced the biodegradation of the nanomaterials. Furthermore, S^2−^ ions from CuS nanodots of LDH-CuS NCs could be easily oxidized upon endocytosis in TME under 808 nm laser irradiation, which accelerated the degradation of nanomaterials. Later, a large amount of Cu^+^ ions would be released from CuS nanodots and accelerated nanocrystals degradation [30]. In a Fenton-like reaction, the free Cu^+^ could efficiently catalyze the conversion of H_2_O_2_ to ·OH. Extensive subcellular ROS were generated in situ by accumulating LDH-CuS NCs in lysosomes, leading to lysosomal membrane permeabilization pathway-associated cell death. In the in vivo antitumor study, the tumor growth in LDH-CuS-NCs laser (−) group was inhibited for the first 2 d post-injection due to the chemodynamic effect of Cu^+^. However, the tumor continued to grow when LDH-CuS NCs were degraded and eliminated from mice. The better therapeutic efficacy for LDH-CuS NCs-laser (+) group compared with LDH-CuS NCs-laser (−) was due to the high temperature-assisted CDT. Furthermore, LDH-CuS NCs-laser (−) group showed a 40% survival rate with 36 days post-treatment, whereas the survival rate of the LDH- CuS NCs-laser (+) group remained 100%.

In another study, a kind of PVP-modified CuS nanocrystals (CuS NCs) with high photothermal conversion efficiency and acidic environment/near-infrared (NIR) light-triggered degradation properties was considered as a promising nanotheranostic platform for synergistic photothermal and CDT therapy (Fig. 13b) [52]. Under the acidic TME and 808 nm laser irradiation, a large amount of Cu^+^ ions were released from CuS NCs and accelerated the degradation of nanocrystals. The released Cu^+^ ions generated ROS for tumor destruction. After 16 days, the tumor growth of mice in the CuS + NIR group was completely suppressed compared with that of the control group. The tumor tissues in the CuS + NIR group were necrotic. Moreover, the mice in the CuS + NIR group were still alive after 22 days of treatment, while all mice in the control group were dead, indicating the promising antitumor effect of CuS NCs. In general, the degradable and biocompatible MeSNs have promising ROS generation and tumor suppression effect.

## Gas therapy

Known for its causticity, H_2_S is a highly toxic gas that is flammable and has a distinct rotten egg odor [127, 128]. Endogenous H_2_S, in addition to carbon monoxide and nitric oxide, however, is the third major gasotransmitter [129–131]. As a biological gaseous signaling molecule, H_2_S plays a key role in different pathological and physiological processes like cancer, diabetes, and Alzheimer’s disease [132, 133]. Researchers established a new therapeutic modality based on the bioeffects of H_2_S, referred to as gas therapy for cancer treatment [134–136]. Endogenous H_2_S generation can be catalyzed by H_2_S-producing enzyme [137]. Pro-cancer effects can result from low levels of H_2_S while cancer inhibition can be induced by high H_2_S levels [138]. Oxidative stress accumulated in cancer cells can also result from excessive H_2_S due to the suppression of the enzyme catalase (CAT) [139] which is recognized as the most vital enzyme for the decomposition of H_2_O_2_ [14].

**Fig. 14:**
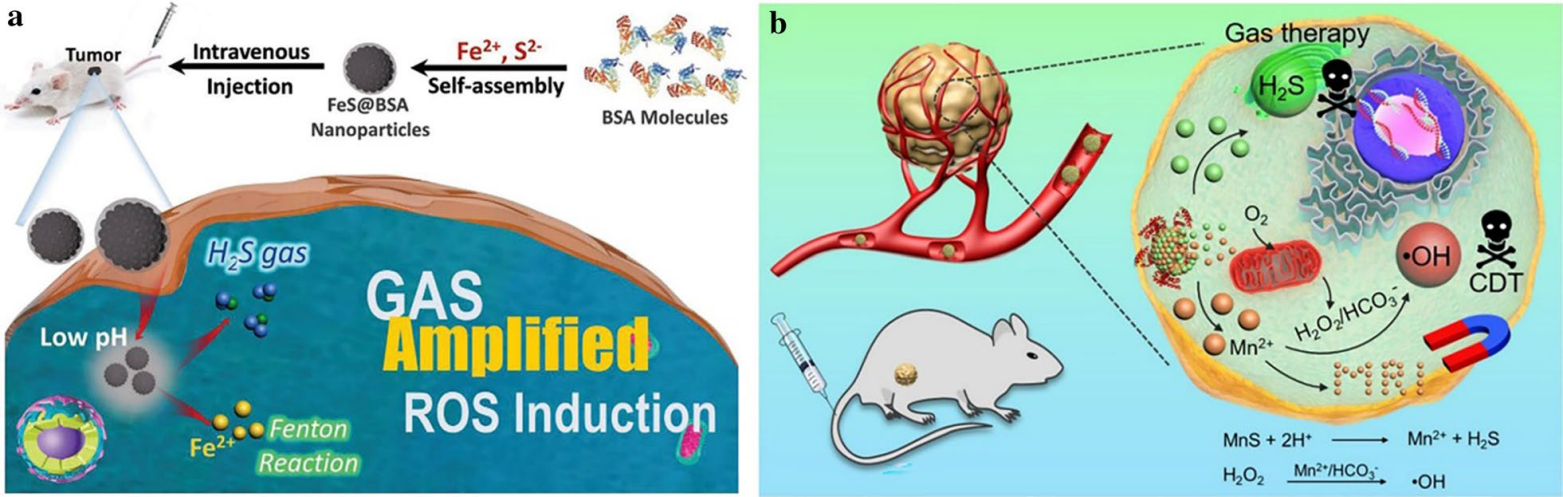
Schematic illustration of therapeutic process of FeS@BSA nanoclusters (**a**). Reprinted with permission from ref [14]. Copyright 2020 Wiley-VCH. Metastable-phase MnS@BSA for tumor pH-responsive traceable H_2_S gas therapy primed CDT of cancer (**b**) (Reprinted with permission from Ref. [64]. Copyright 2020 IVY Publisher)

Interestingly, the release of S^2−^ during the degradation process in the acidic microenvironment of tumors is another fascinating feature of MeSNs. H_2_S gas is produced when one S^2−^ ion combines with two protons in TME. Thus, gas therapy is another anti-tumor strategy which could be achieved by the degradation products of MeSNs other than CDT. Xie and co-workers took the first attempt of the metal sulfide nanomaterial-based tumor gas therapy (Fig. 14a) [14]. In their research, the synthesis of amorphous ferrous sulfide embedded bovine serum albumin (FeS@BSA) nanoclusters was achieved via a self-assembly approach. The nanoclusters were degraded under acidic conditions and released Fe^2+^ ions and H_2_S gas simultaneously. A specific suppression effect was produced by the released H_2_S on the CAT activity of cancer cells, resulting in H_2_O_2_ facilitating the Fenton reaction of Fe^2+^ and consequently promoting ROS induction within the cells. During the 14 days of treatment, mice treated with PBS showed fast-growing tumors, and a certain inhibition was observed in Na_2_S or Fe^2+^@BSA solutions treated groups. Na_2_S and Fe^2+^@BSA presented moderate inhibition rates of ~ 27 wt% and ~ 50 wt%, respectively. In contrast, tumor growth was remarkably suppressed in mice injected with the dispersion containing FeS@BSA nanoclusters. The maximum tumor inhibition rate of ~ 71 wt% indicated its excellent anti-tumor properties, which were contributed by the synergetic effect of Fe^2+^ and H_2_S.

In another study, He et al. constructed a type of nanotheranostics (MnS@BSA) by embedding BSA with MnS NPs (Fig. 14b) [64]. In response to the mildly acidic TME, the as-prepared MnS@BSA underwent degradation and generated ROS by releasing H_2_S which inhibited the activity of CAT. Moreover, the released Mn^2+^ presented strong signals for magnetic resonance imaging (MRI) and achieved MRI-guided cancer therapy. In the in vivo antitumor study, the saline group showed fast tumor growth, whereas the MnS@BSA treated group exhibited higher tumor suppression compared to MnCl_2_ and Na_2_S treated groups. The life expectations of mice administered with MnS@BSA were greatly prolonged among the above groups. The above studies have opened new horizons for traceable H_2_S gas primed CDT of cancer.

The MeSNs mentioned above achieved gas therapy by inhibiting the activity of CAT enzyme. However, as an endogenous signaling molecule, the roles of H_2_S in physiological and pathological processes are complex and diverse. Recently, Yang et al. reported that the H_2_S gas released from PVP modified multifunctional iron sulfide nanoparticles (Fe_1 − x_S-PVP NPs) could suppress the activity of enzyme cytochrome C oxidase of cancer cells and inhibit the tumor growth [140]. Furthermore, H_2_S could affect the function of mitochondrial through irreversible oxidation by sulfide-quinone oxidoreductase [135]. Up to now, the research on H_2_S-based gas therapy has just begun, and extensive studies are needed to clarify the mechanism of actions.

## Immunotherapy

**Fig. 15:**
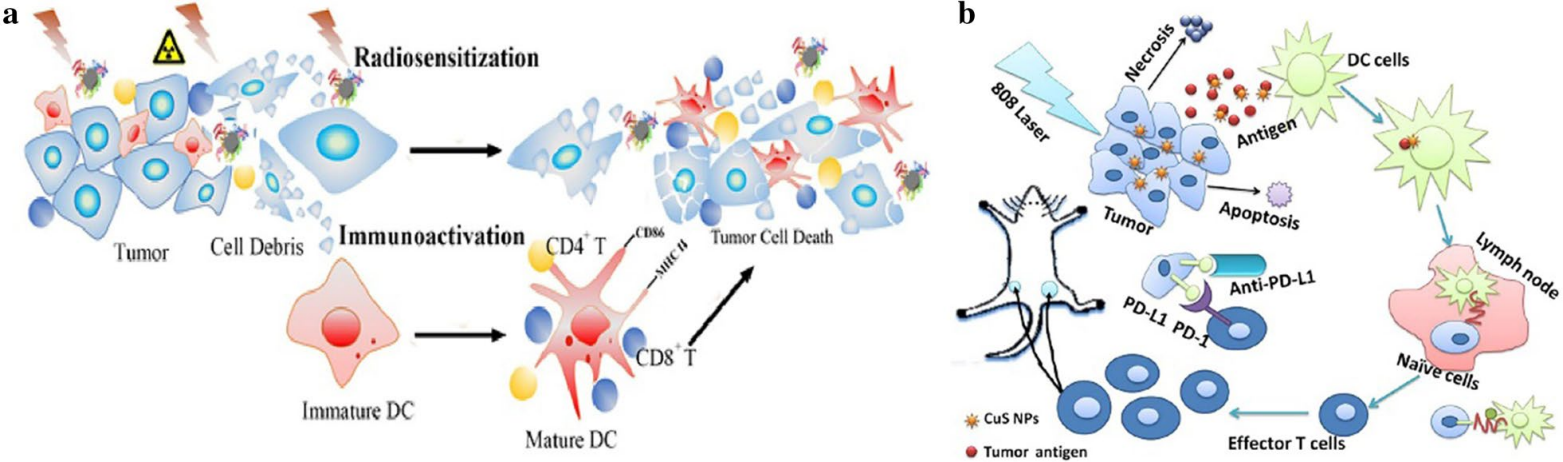
Schematic illustration of GLP-BiNP for radiosensititive dendritic cell activation for cancer therapy (**a**). Reprinted with permission from ref [15]. Copyright 2019 American Chemical Society. Schematic diagram of anti-tumor immune responses induced by CuS NPs-PEG-Mal (**b**) (Reprinted with permission from Ref. [44]. Copyright 2019 American Chemical Society)

Considering that phototherapy and RT can only be applied in managing local tumors, immunotherapy as a systemic therapy has been gradually integrated with other therapeutic strategies for better antitumor effect. Dendritic cell (DC) is a typical cell type for antigen presenting. DC has been considered as the most important targeted cell type since the first published clinical trials in the mid-1990 s, and DC-based immunotherapy was approved by the US FDA in 2010 [15]. However, the function of DC maturation and the number of effective T cells are significantly suppressed by cancer cells. Fortunately, radiation can reverse the above phenomena by changing the TME and triggering the immunotherapy [141]. Yu and co-workers found that DC could be mildly activated by a kind of Bi_2_S_3_ NPs (BiNP) alone in vitro, while the level of DC maturation was further enhanced by Ganoderma lucidum polysaccharide (GLP, with immunoactivity) conjugated BiNP (GLP-BiNP), and manifested as the increase in cytokine release, phenotypic maturation markers, acid phosphatase activity, and T cell proliferation in DC/T cell co-culture (Fig. 15a) [15]. GLP-BiNP treatment alone seemed to have a partial inhibitory effect on tumor growth, likely attributed to the immunostimulatory response of GLP and the adjuvant effect of nanoparticles. X-ray alone could inhibit tumor growth, while the inhibitory effects were further enhanced, and statistically significant differences were showed when X-ray was combined with BiNP or GLP-BiNP. It was worth noting that GLP-BiNP combined with X-ray completely prevented the tumor growth compared to BiNP plus X-ray group. For the future perspective of immunotherapy, the strategy to incorporate the immunoactivity polysaccharides into MeSNs may hold great potential for tumor treatment.

Following photothermal ablation of the tumor, generation of an antigen associated with the tumor in situ can give rise to a vaccine-like effect and stimulate an immune response in vivo. Wang et al. constructed a kind of CuS NPs that were not only used as a photothermal mediator for tumor hyperthermia but also as an antigen-capturing agent to induce tumor response during hyperthermia via absorbing tumor antigens (anti-PD-L1) (Fig. 15b) [44]. In combination with immune checkpoint blocker, the engineered NPs (CuS NPs-PEG-Mal) modified with maleimide PEG and bearing a stronger antigen adsorption capacity were used to evaluate the effect of hyperthermia improving immunotherapy in the 4T1 breast cancer tumor model. The in vivo studies depicted that CuS NPs-PEG-Mal based hyperthermia resulted in a distinct rise in the serum levels of inflammatory cytokines, leading to the immunogenic TME. PTT mediated by CuS NPs-PEG-Mal, in cooperation with anti-PD-L1, increased the amount of tumor-infiltrating CD8^+^ T cells and resulted in inhibition of the growth of distant as well as primary tumors in the 4T1 tumor model. Tumors in mice that received CuS NPs-PEG-Mal-containing adsorption protein antigens clearly showed significantly slower growth. However, no appreciable tumor growth inhibitory effect was observed in the PBS plus anti-PD-L1 groups, which indicated that only CuS-NPs-PEG-Mal adsorbing protein antigens could stimulate the immune system and inhibit the tumor growth.

## Combination therapy

**Fig. 16:**
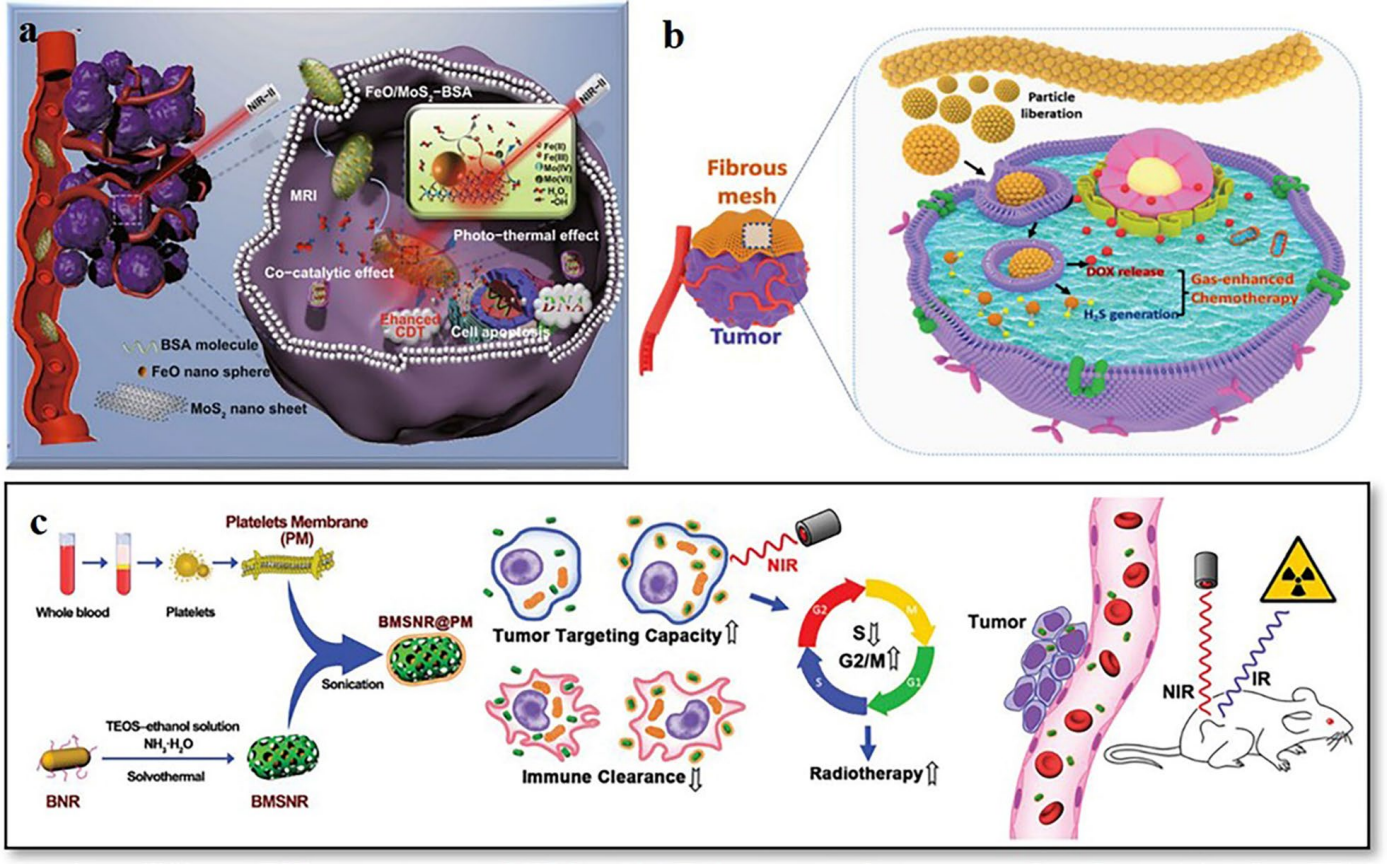
Schematic presentation of FeO/MoS_2_-BSA for MRI and synergetic enhanced CDT/PTT (**a**). Reprinted with permission from ref [142]. Copyright 2020 Springer. Schematic illustration of DOX-ZnS@SiO_2_ fibrous mesh for localized H_2_S-amplified chemotherapy (**b**). Reprinted with permission from ref [21]. Copyright 2020 Royal Society of Chemistry. Schematic illustration of BMSNR@PM for PTT/RT synergistic therapy (**c**) (Reprinted with permission from Ref. [49]. Copyright 2019 Royal Society of Chemistry)

The functional classification of MeSNs in this review makes it easier to understand the roles of intact nanoparticles, metal ions, and sulfide ions in cancer therapy respectively. In many cases, bioactive MeSNs can exert multiple anti-tumor effects (Table 2). Nanosized MeSNs can be used as drug carriers to achieve chemotherapy, while MeSNs with phototransformation ability can be used for phototherapy. After the degradation in the tumor environment, the released metal ions and S^2−^ can activate CDT, gas therapy, or immunotherapy. Zhang et al. reported a kind of BSA-modified FeO/MoS_2_ nanocomposites (FeO/MoS_2_-BSA) with boosted Fenton reaction efficiency resulted from the synergistic effect of metal catalysts and the photothermal effect of MoS_2_ nanosheets triggered by the second NIR light (Fig. 16a) [142]. In the TME, the Mo^4+^ on the surface of MoS_2_ nanosheets not only accelerated the conversion of Fe^3+^ to Fe^2+^ and improved Fenton reaction efficiency but also endowed FeO/MoS_2_-BSA with good photothermal performances for photothermal-enhanced CDT and PTT. The tumor growth after the treatment of FeO/MoS_2_-BSA nanocomposites was obviously slower than those of the control group and laser group, which could be ascribed to the good co-catalytic effect of MoS_2_ and FeO for CDT. In contrast, it was found that the tumors treated with FeO/MoS_2_-BSA nanocomposites and exposed to 1064 nm laser were thoroughly ablated on the 5th day without recurrence within 14 days, indicating the excellent anticancer effect of synergistic CDT and PTT. In another study, localized H_2_S-amplified chemotherapy was achieved via ZnS nanoparticle-decorated silica fiber mesh [21]. Implanted DOX loaded ZnS NPs assembled silica fibres (DOX-ZnS@SiO_2_) enabled sufficient on-site drug dosage and intracellular H_2_S contents (Fig. 16b). The released DOX and H_2_S showed significant synergistic tumor inhibition. The tumor progression of mice treated with a low dose of free DOX was partially inhibited. Mice treated with ZnS@SiO_2_ fibres showed considerable tumor suppression compared with the free DOX group. However, for those treated with DOX-ZnS@SiO_2_ fibres, the tumor shrank after 14 days of treatment. Furthermore, H&E-stained microscopy slices of tumors indicated that the tumor tissues treated with DOX-ZnS@SiO_2_ fibres were more seriously damaged than those with free DOX. However, in the ZnS@SiO_2_ fibres treated group, the tumor tissues retained their pathological state. This study proved that gas therapy combined with chemotherapy could exert a more powerful anti-tumor effect. Furthermore, cancer cells were efficiently eradicated after treating with the platelet membrane-camouflaged bismuth-containing nanorods (BMSNR@PM) due to the combined action of RT and PTT in vivo, thereby remarkably enhancing the survival of 4T1 tumor-bearing mice (Fig. 16c) [49]. In general, although the composition of MeSNs is simple, each component of MeSNs can exert a different anti-tumor effect. Having diverse synergistic therapeutic effects is the biggest advantage of MeSNs over other drug delivery systems. Researchers can construct multifunctional anti-tumor MeSNs according to the intended treating purposes.

## Conclusion and perspectives

Metal elements play an important role in the field of tumor treatment. With the development of nanotechnology, metal-containing nanomaterials can further overcome defects of metal compounds, such as short circulation time, little discrimination between tumor and healthy tissues, and dose-limiting systematic toxicity. Moreover, the nanosized metal-containing nanoparticles can exhibit special physical and chemical properties, such as Fenton reaction catalysis, light conversion, and radiation enhancement, which has attracted the attention of researchers for cancer therapy. The purpose of this review is to outline the latest advances of MeSNs for clarifying the developing direction and promoting the clinical transformation of metal sulfide nanocomposites. The first section of this review summarizes the preparation approaches used for medical application and analyzes the differences and advantages of the various approaches. This part aims to guide researchers to choose more suitable approaches to prepare the desired MeSNs. The second section of this article sorts out the anti-tumor effects and mechanisms of different MeSNs. It is worth emphasizing that the intact MeSNs can achieve energy conversion for phototherapy and radiotherapy, while metal ions and H_2_S will be generated during their degradation for CDT and gas therapy, etc. Therefore, MeSNs usually exhibit synergistic antitumor properties, which is the biggest advantage of MeSNs compared to other nano-therapeutic agents.

Although MeSNs-induced cancer therapy strategies have undergone rapid development, the discovery of their therapeutic effects is still in infancy. Specifically, the treatment efficacy of the MeSNs may rely on their intrinsic parameters, such as the accumulation efficacy within the tumor site. Hence, more endeavors should be made to explore the functionalization of MeSNs with specific target molecules for tumor accumulation. Secondly, additional attention should be paid to the biosafety of MeSNs. Most of the existing studies focused on the anti-tumor effects and mechanisms of MeSNs. The degradation and metabolism of MeSNs in the body have not been clarified. Non-specific degradation products of MeSNs, such as metal ions and highly toxic ROS, are likely to affect the metal metabolism and damage the physiological functions of normal tissues. Thus, further studies characterizing the short and long-term fate of the MeSNs in vivo should be done to prompt the translation process, such as biodistribution studies, pharmacokinetic studies, metabolism studies, and elimination studies. Moreover, the development of easy preparation methods and scale-up will advance the reproducibility of the MeSNs. Finally, an elaborate clarification regarding the clinical effectiveness of MeSNs is much needed. The MeSNs-interference therapy strategies are still in the initial stage of exploration, and almost all research data come from animals only. Extensive research is continuously required to validate its effectiveness. In general, although MeSNs exhibit excellent anti-tumor effects, their applications in the field of life sciences are still immature. Therefore, multidisciplinary efforts from biologists, chemists, and materials scientists are needed to obtain a clear understanding of MeSNs-based nanomedicine, which will further facilitate the clinical translation. We believe that with the advancements in research, MeSNs will have an important impact on future cancer treatments.

## Data Availability

Not applicable.

## Abbreviations

MeSNs: Metal sulfide nanomaterials
Pt: Platinum
Ag_2_S: Silver sulfide
CuS: Copper sulfide
NPs: Nanoparticles
NIR: Near-infrared region
Bi: Bismuth
Bi_2_S_3_: Bismuth sulfide
MnS: Manganese sulfide
FeS: Iron sulfide
ZnS: Zinc sulfide
TEM: Transmission electron microscope
SEM: Scanning electron microscope
TME: Tumor microenvironment
H_2_S: Hydrogen sulfide
ROS: Reactive oxygen species
PVP: Polyvinylpyrrolidone
PEG: Polyethylene glycol
MoS: Molybdenum sulfide
CTAC: Cetyltrimethylammonium chloride
BSA: Bovine serum albumin
SnS: Tin sulfide
TaS_2_: Tantalum sulfide
WS_2_: Tungsten sulfide
CdS: Cadmium sulfide
*S. oneidensis*: *Shewanella oneidensis*
FeOOH NSs: Iron oxide-hydroxide nanospindles
VSx: Vanadium sulfide
PDA: Polydopamine
NiS: Nickel sulfide
Hm-NiS: Hollow mesoporous nickel sulfide nanoparticles
DOX: Doxorubicin
H-CuS NPs: Hollow CuS nanoparticles
Ce6: Chlorin e6
SnS NSs: Tin sulfide nanosheets
PTT: Photothermal therapy
PDT: Photodynamic therapy
^1^O_2_: Singlet oxygen
Bi_2_S_3_ NRs: Bi_2_S_3_ nanorods
ZP: Zinc protoporphyrin IX
BPZP: Bi_2_S_3_ NR-P(NIPAM-*co*-AM)-ZP-Pep nanosystems
RT: Radiotherapy
CDT: Chemodynamic therapy
H_2_O_2_: High hydrogen peroxide
OH: Hydroxyl radicals
LDH-CuS NCs: Layered double hydroxide-copper sulfide nanocomposite
CAT: Catalase
MRI: Magnetic resonance imaging
ZnS@SiO_2_: ZnS nanoparticle-decorated silica fiber mesh
Fe_1−x_S-PVP NPs: PVP modified multifunctional iron sulfide nanoparticles
DC: Dendritic cell
BiNP: Bi_2_S_3_ NPs
GLP: Ganoderma lucidum polysaccharide
